# A precise molecular subtyping of ulcerative colitis reveals the immune heterogeneity and predicts clinical drug responses

**DOI:** 10.1186/s12967-023-04326-w

**Published:** 2023-07-13

**Authors:** Shaocong Mo, Bryan Jin, Yujen Tseng, Lingxi Lin, Lishuang Lin, Xin Shen, Huan Song, Mingjia Kong, Zhongguang Luo, Yiwei Chu, Chen Jiang, Zhiwei Cao, Jie Liu, Feifei Luo

**Affiliations:** 1 Department of Digestive Diseases, Huashan Hospital, Fudan University, 12 Wulumuqi Middle Road, Shanghai, 200040 China; 2 National Clinical Research Center for Aging and Medicine, Huashan Hospital, Fudan University, Shanghai, 200040 China; 3 Department of Pathology, Huashan Hospital, Fudan University, Shanghai, China; 4 Biotherapy Research Center, Department of Immunology, School of Basic Medical Sciences and Institute of Biomedical Sciences, Fudan University, Shanghai, 200032 China; 5 Department of Pharmaceutics, School of Pharmacy, Fudan University, Shanghai, 201203 China; 6 School of Life Sciences, Fudan University, Shanghai, 200433 China; 7 School of Life Sciences and Technology, Tongji University, Shanghai, 200092 China

## Abstract

**Background and Aims:**

We sought to identify novel molecular subtypes of ulcerative colitis (UC) based on large-scale cohorts and establish a clinically applicable subtyping system for the precision treatment of the disease.

**Methods:**

Eight microarray profiles containing colon samples from 357 patients were utilized. Expression heterogeneity was screened out and stable subtypes were identified among UC patients. Immune infiltration pattern and biological agent response were compared among subtypes to assess the value in guiding treatment. The relationship between PRLR and TNFSF13B genes with the highest predictive value was further validated by functional experiments.

**Results:**

Three stable molecular subtypes were successfully identified. Immune cell infiltration analysis defined three subtypes as innate immune activated UC (IIA), whole immune activated UC (WIA), and immune homeostasis like UC (IHL). Notably, the response rate towards biological agents (infliximab/vedolizumab) in WIA patients was the lowest (less than 10%), while the response rate in IHL patients was the highest, ranging from 42 to 60%. Among the featured genes of subtypes, the ratio of PRLR to TNFSF13B could effectively screen for IHL UC subtype suitable for biological agent therapies (Area under curve: 0.961–0.986). Furthermore, we demonstrated that PRLR expressed in epithelial cells could inhibit the expression of TNFSF13B in monocyte-derived macrophages through the CXCL1-NF-κB pathway.

**Conclusions:**

We identified three stable UC subtypes with a heterogeneous immune pattern and different response rates towards biological agents for the first time. We also established a precise molecular subtyping system and classifier to predict clinical drug response and provide individualized treatment strategies for UC patients.

**Supplementary Information:**

The online version contains supplementary material available at 10.1186/s12967-023-04326-w.

## Introduction

Ulcerative colitis (UC) is one of the two types of inflammatory bowel diseases (IBD), characterized by chronic inflammation from the rectum to the proximal colon [1]. UC is a huge economic burden worldwide, which generates an estimated total expenditure of nearly US$10 billion per year in the USA and €30 billion in Europe [2].

Five-aminosalicylic acid, corticosteroids, thiopurines, anti-tumor necrosis factor (TNF) agents, anti-adhesion therapy/anti-integrins, calcineurin Inhibitors, and Janus Kinase Inhibitor therapy all contribute to the clinical remission of UC [3, 4]. However, drug resistance, disease recurrence, and adverse effects of the medication are major factors that lead to poor clinical outcomes in patients. For biological agents such as Infliximab and Vedolizumab, the total response rate of patients was less than 40% [5–7], which indicates possible heterogeneity among patients. Herein, it is urgent to elucidate the subclassing of UC to avoid confounding therapies. However, only limited studies concentrated on the molecular subtyping of UC. Intriguingly, it has been discovered that the mucosa of Crohn’s disease (CD) in adults could be divided into normal-like and ileum-like subtypes, which differed in surgical treatment incidence, suggesting the significance of IBD subtyping [8]. In contrast, molecular sub-classifications of UC have not been reported yet.

To establish a molecular subtyping system for UC, we first identified the heterogeneity via consensus clustering based on training UC colon samples from public datasets. Three stable subtypes of UC: Innate Immune Activated (IIA), whole Immune Activation (WIA), and Immune Homeostasis Like (IHL) were defined using CrossICC algorithm and immune cell infiltration evaluation. IHL was identified to have the best response rate to biological agents among the three subtypes. We further discovered that the expression ratio of prolactin receptor (PRLR) to tumor necrosis factor superfamily member 13b (TNFSF13B) could facilitate IHL identification. The underlying mechanism was that PRLR in epithelial cells inhibited CXCL1 secretion and the NF-κB-TNFSF13B axis in macrophages to attenuate inflammatory reactivity. Collectively, this is the first molecular classification system established for UC to optimize the selection of biological agent therapy, which is potentially translatable to clinical practice.

## Materials and methods

### Data resources and clinical samples

**Fig. 1:**
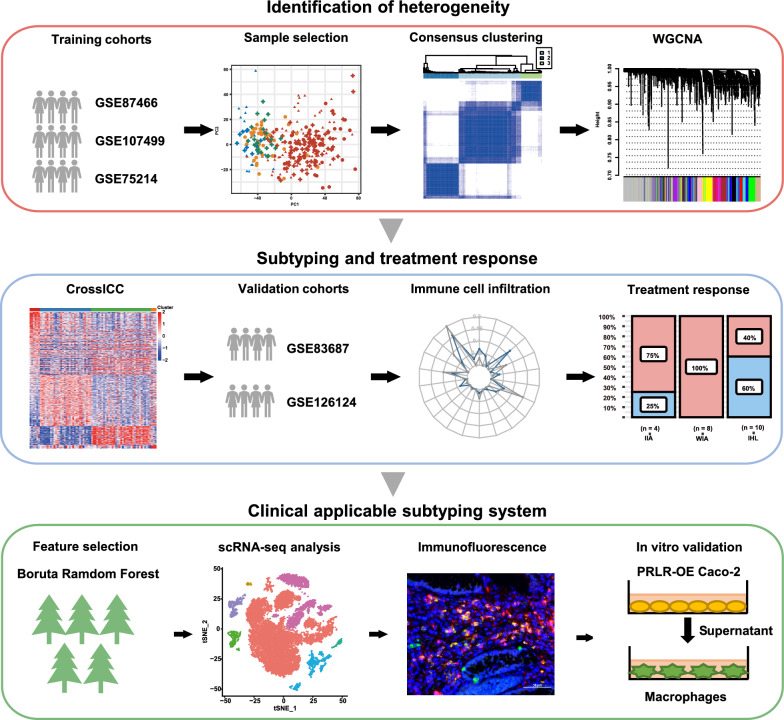
Workflow of the study

In the current study (Fig. 1), datasets of UC were obtained from Gene Expression Omnibus (GEO). After selection, GSE87466, GSE107499, and GSE75214 were adopted as training cohorts. GSE83687 and GSE126124 were used as validation cohorts while GSE114527, GSE73661, and GSE16879 were used to compare the drug responses, in which samples were collected prior to treatment [9–15]. For the included datasets, only colon tissue samples were included for analysis. The expression profiles all underwent log2 transformation. Two single-cell RNA-sequencing (scRNA-Seq) datasets, GSE182270 and GSE150115 were applied to determine cell types in which feature genes were predominantly expressed [16, 17], which underwent Seurat standard pipeline. Information of the included datasets is listed in detail (Additional file 1: Table S1).

Twelve UC patients with active disease were enrolled in Huashan Hospital, Fudan University, Shanghai from September 2021 to Feburary 2023. Prior to biological agent treatment, the mucosal samples were collected and stored at -80 °C protected by RNA-later for RNA sequencing or underwent formalin fixation and paraffin embedding for immunofluorescence analysis. Patients received repeat colonoscopy after 8 weeks of biological agent treatment. Treatment response was defined as a total Mayo score less than or equal to 2, with no subscore larger than 1 and a rectal bleeding subscore equal to 0 at 8 weeks (Table 1, Additional file 1: Table S2). Informed consent was obtained from all the patients included in the present study. The detection of the human tissues involved in the study was received ethical approval of the Ethical Committee of Medical Research, Huashan Hospital of Fudan University (No 2013-005).

**Table 1:** Information of the patients in FDUHS cohort

Patients	Gender	Age	Treatment	Response	Subtype
P1	Male	24	Vedolizumab	NR	IIA
P2	Male	59	Vedolizumab	NR	IHL
P3	Female	49	Vedolizumab	NR	WIA
P4	Male	31	Vedolizumab	R	IHL
P5	Female	39	Vedolizumab	NR	WIA
P6	Female	42	Infliximab	NR	WIA
P7	Female	54	Infliximab	R	IHL
P8	Female	34	Infliximab	R	IHL
P9	Male	40	Vedolizumab	NR	WIA
P10	Male	43	Vedolizumab	NR	WIA
P11	Male	63	Vedolizumab	R	IHL
P12	Male	62	Vedolizumab	NR	IHL

### Principal component analysis (PCA) consensus clustering

PCA was employed for sample selection via prcomp function. For the sample selection, only pathological tissues from patients with active disease which were distanced from the normal mucosa in PCA were included. Consensus clustering was used to preliminarily discover the heterogeneity of UC with ConsensusClusterPlus package [18]. Common genes were intersected and batch effect was removed with Combat function for samples prepared for consensus clustering. Clusters were yielded by k-means and Euclidean distance. Consensus score was then calculated. The optimal number of clusters was considered to have a score higher than 0.8 for each cluster. To generate a precise classification, the number of clusters should be as many as possible. Differentially expressed genes (DEGs) were partitioned into DEGs for each subtype compared with normal samples and DEGs for each subtype compared with other subtypes. Genes with log2 mean difference which was larger than 0.2 and FDR less than 0.05 were considered as DEGs [19].

Stable subtypes were finally identified by CrossICC, which is a novel subtyping algorithm that avoids the confounding effect in the process of removing batch effect [20]. Subtypes of external validation datasets could be predicted with the predictor function of CrossICC.

### Functional enrichment and WGCNA

Biological process of Gene Ontology (GO) and Kyoto Encyclopedia of Genes and Genomes (KEGG) analysis was applied for the functional annotation via clusterProfiler package [21]. The feature genes identified by consensus clustering underwent WGCNA [22]. Hierarchical clustering was conducted on the samples to remove abnormal ones. Soft threshold (power value) was determined when the scale-free fit index (signed R2) reached 0.85 to ensure that the relationship of the genes reached a scale-free network. The feature genes were ultimately clustered into different gene modules for the downstream analysis.

### Evaluation of immune cell infiltration and gene signature

CIBERSORT (R version 1.03) was utilized to evaluate the infiltration of 22 types of immune cells of each UC sample [23]. Subsequently, samples with *P* value larger than 0.05 were removed to ensure a reliable evaluation. In order to calculate the absolute infiltration score of the immune cells, single sample gene set enrichment analysis (ssGSEA) was applied with the gene sets extracted from a recent study via GSVA package with gene sets obtained from a past research [24, 25].

Gene sets to evaluate the epithelial functions and therapeutic targets of UC were obtained from GSEA-MSigDB (http://www.gsea-msigdb.org/). GSVA package was also employed to calculate the enrichment score of each sample. For therapeutic targets, classic targets leukotriene and thrombin, prostaglandin and platelet were included. TNF, integrin and Janus kinase pathway inhibitors were included as advanced targets (Additional file 1: Table S3).

### Dimensional reduction and identification of dependent biomarkers

Boruta package was employed for the dimension reduction which applied the random forest algorithm to extract features and the order of them was scrambled to calculate the importance of features [26]. Setting the maximal number of importance source runs to 100 and the tree numbers to 500, confirmed variates were left for tenfold cross-validated Least Absolute Shrinkage and Selection Operator (LASSO) regression. The expressions of included genes were scaled in each sample. Ten-fold cross-validated Support Vector Machine (SVM) was applied to verify the significance of the 16 genes with best cost and gamma value set using e1071 package. To identify the smallest number and concise crucial biomarkers which could accurately distinguish the subtypes, genes with the absolute value of the coefficient larger than 1 were selected to calculate the expression ratio.

### RNA-sequencing (RNA-seq)

The total RNA of the clinical UC tissues and cell lines was extracted by TRIzol Reagent (Invitrogen, California, USA). The total amount of RNA was quantified by NanoDrop ND2000 (Thermo Fisher Scientific, Massachusetts, USA). cDNA library was built by TruSeq RNA sample preparation Kit (Illumina, San Diego, CA, USA) and the fragment sequencing was conducted on Illumina hiseq3000. Quality control of the sequencing results was done by fastQC and the data was filtered via trim_galore, after which the sequencing was aligned into Homo sapiens GRCh38 with Hisat2, quantified by featureCounts. The RNA-sequencing was performed in Neo Bio-technology (Shanghai, China).

### Immunofluorescence

Paraffin-embedded colonic mucosa sections first underwent deparaffinization and antigen retrieval and was then blocked with 5% BSA for 30 min. Then, the section was incubated with anti-PRLR (Abcam, ab170935, Cambridge, UK, 1: 250) and anti-TNFSF13B (Abcam, ab203791, 1: 100), and anti-CD11b (Abcam, ab52478, Cambridge, UK, 1: 250) at 4 °C overnight, with antibodies diluted in primary antibody dilution buffer (Servicebio, Wuhan, China). Subsequently, the tissues were washed by PBS for 3 times and incubated with fluorescent-labeled secondary antibody diluted in PBS at room temperature for 45 min (Servicebio, GB21303, 1:300; GB25303, 1: 400). Finally, DAPI staining was conducted at room temperature for 20 min. Fluorescence microscope (Olympus IX73, Tokyo, Japan) was used to observe the fluorescent images.

### Cell line

Human intestinal epithelial cell line Caco-2 and human monocyte cell line THP-1 were purchased from ATCC (Manassas, Virginia, USA). Caco-2 was cultured in Dulnecco’s Modified Eagle’s Medium (DMEM), supplemented with 20% fetal bovine serum (FBS; Gibco, New York, USA) and penicillin (100 U/mL)-streptomycin (100 U/mL). THP-1 was cultured in Roswell Park Memorial Institute (RPMI) 1640 medium supplemented with 10% FBS. For the induction of THP-1 monocytes to macrophages, cells were treated by phorbol 12-myristate 13-acetate (PMA) (Sigma-Aldrich, Missouri, USA) for 48 h. To mimic the in vivo inflammatory status of colon, Caco-2 was stimulated with 1 μg/mL Lipopolysaccharide (LPS) for 48 h. Then, the culture supernatant was collected and added to THP-1 macrophages [27]. To antagonize CXCR2 of THP-1, the sole receptor of CXCL1, THP-1 cells were pretreated with CXCR2 antagonist SB225002 for 30 min (Selleck, Texas, USA, 10 μM). To inhibit NF-κB of THP-1, we used NF-κB inhibitory Bay 11-7082 to pretreated THP-1 cells for 30 min (Sigma-Aldrich, 1 μM).

### Plasmid construction, lentivirus production and cell infection

To construct cell line that overexpressed PRLR, the whole length cDNA of PRLR (NM_000949) was cloned into GV492 (Ubi-MCS-3FLAG-CBh-gcGFP-IRES-puromycin) (Genechem, Shanghai, China). Primers for PRLR were used as following:

Forward primer (5′ − 3′): AGGTCGACTCTAGAGGATCCCGCCACCATGAAGGAAAATGTGGCATC, Reverse primer (5′ − 3′): TCCTTGTAGTCCATACCGTGAAAGGAGTGTGTAAAACATG.

GV492 containing PRLR cDNA (20 μg), packaging plasmid pHelper 1.0 (15 μg) along with pHelper 2.0 (10 μg) were co-transfected into 293 T. After 6 h, medium was discarded and fresh medium was added. The supernatant of 293 T cells was collected 48 h after transfection and underwent ultracentrifuged and resuspend. Subsequently, Caco-2 cell line was transfected by the lentivirus containing PRLR (Titer = 1E + 9 TU/ml, MOI = 10, HitransG P Infection Enhancer (Genechem)), and then further selected by puromycin. Empty GV492 was used as negative control lentivirus.

### Quantitative real-time PCR (qRT-PCR)

Total RNA of cell lines was extracted by TRIzol (Invitrogen). Complementary DNA was acquired using Hifair III 1st Strand cDNA Synthesis SuperMix for qPCR (gDNA digester plus) (Yeasen, Shanghai, China). qRT-PCR was conducted with the Hieff qPCR SYBR Green Master Mix (Yeasen) according to the manufacturer's instructions. Subsequently, relative transcript expression was calculated by the ΔΔCt method, with ACTB (β-actin) applied as the endogenous reference. The primer sequences used in the qRT-PCR are as following:

PRLR Forward primer (5′ − 3′): TCTCCACCTACCCTGATTGAC, PRLR Reverse primer (5′ − 3′): CGAACCTGGACAAGGTATTTCTG.

BAFF Forward primer (5′ − 3′): GGGAGCAGTCACGCCTTAC, BAFF Reverse primer (5′ − 3′): GATCGGACAGAGGGGCTTT.

### Western blotting

Total protein was lysed in RIPA (Thermo Fisher Scientific) along with protease and phosphatase inhibitor cocktail, quantified by BCA Assay. Protein samples were separated on non-reducing SDS-PAGE 12% Tris–HCl gels (Beyotime, Shanghai, China) and then transferred onto PVDF membranes. The membrane was then blocked in 5% skimmed milk for 1 h, after which the membrane was incubated with primary antibodies diluted in primary antibody dilution buffer (Beyotime, Shanghai, China) overnight at 4 °C followed by incubation with horseradish peroxidase-conjugated secondary antibodies. Blots were developed with chemiluminescent detection reagent and imaged with a Chemiluminescent Imaging System (Tanon, Shanghai, China). Western blot quantification was conducted using ImageJ. The western blot antibodies, were listed as follows: anti-PRLR (Abcam, ab170935, 1: 1,000), anti-TNFSF13B (Abcam, ab224710, 1: 1,000), anti-GAPDH (Cell Signaling Technology, 5174, Massachusetts, USA, 1: 1,000), anti-NF-κB p65 (Cell Signaling Technology, 8242, 1: 1,000), anti-Phospho-NF-κB p65 (Ser536) (Cell Signaling Technology, 3033, 1: 1,000), anti-rabbit IgG (Cell Signaling Technology, 7074, 1: 5,000 in Tris Buffered Saline-Tween).

### Statistical analysis

Wilcoxon test was used for the comparison of continuous variables between the two subtypes. Kruskal–Wallis test was used for comparisons among multiple subtypes. Chi-square test and Fisher test were applied for categorical variable. All the statistical analysis was done in R (version 4.0.3).

## Results

### Identification of the heterogeneity of UC

As shown in the study workflow (Fig. 1, panel 2), samples were carefully screened before inclusion to ensure sample quality. PCA was applied to reveal the distribution of the samples in each dataset. For GSE87466, 87 diseased tissues were included (Additional file 1: Figure S1a). For GSE107499, only pathological samples were included (Additional file 1: Figure S1b). For GSE75214, tissues in the active disease state were enrolled (Additional file 1: Figure S1c). Subsequently, batch effect among 3 datasets was removed (Additional file 1: Figure S1d, e). We further demonstrated that the samples (n = 208) included were indeed distinguished from excluded samples (Additional file 1: Figure S1f). Collectively, the samples we included were comparable and evenly distributed in three datasets.

**Fig. 2:**
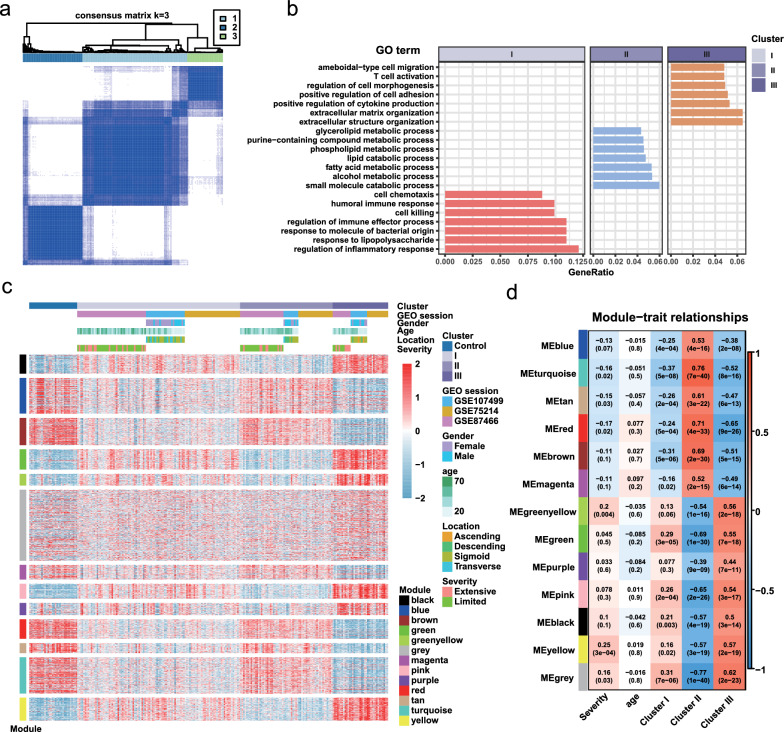
Identification of the heterogeneity of UC. **a** Consensus clustering assigned the samples into 3 clusters. **b** Gene Ontology (GO) enrichment of the DEGs of each cluster. **c** Heatmap of Gene expression classified by modules identified by WGCNA. **d** The module-trait relationship heatmap. **e** Dot plot of the GO enrichment of the genes in each WGCNA module

Then, we sought to identify whether there was distinct heterogeneity amongst the included UC samples. Consensus clustering was applied to the 208 enrolled UC mucosal tissues, which subdivided the samples into 3 clusters. (Fig. 2a, Additional file 1: Figure S2). Additionally, there were no differences in gender, age, or disease severity among the 3 UC clusters (Additional file 1: Table S4), which indicated that the clinical characteristics could not reflect molecular heterogeneity.

The feature genes of each sample were identified for preliminary exploration of the UC clusters. In Cluster I, 95 genes were identified as significantly up-regulated DEGs compared with other clusters, 2619 genes were considered as up-regulated DEGs for Cluster II and 2704 DEGs were up-regulated in Cluster III. Additionally, the biological process of GO enrichment demonstrated that DEGs in Cluster I were enriched in inflammatory and anti-bacterial response, DEGs in Cluster II were mainly involved in metabolic pathways, while DEGs in Cluster III were enriched in the immune and extracellular matrix organization (Fig. 2b).

To obtain a more profound understanding of the DEGs that were identified, WGCNA was employed to analyze the total 5418 genes and 208 included UC samples. Three samples regarded as outliers were removed (Additional file 1: Figure S3a). With soft power set to 13, thirteen gene modules were identified (Additional file 1: Figure S3b, c). The expressions of up-regulated DEGs, data sources, and clinical traits of each cluster along with the WGCNA modules were presented (Fig. 2c, d). There were no statistical differences for various clinical phenotypes among these three clusters, including lesion location and endoscopic severity (Fig. 2c). GO analysis further identified the biological process involvement of the genes in each module, those with statistical significance were marked with a triangle (Additional file 1: Figure S3d). Modules such as black, pink, and green participated in cytokine-cytokine receptor interaction and were closely related to Cluster III, while modules such as red, turquoise, and tan were mainly involved in metabolic pathways and were associated with Cluster II (Additional file 1: Figure S3d, Table S5). Collectively, there were distinct crucial gene modules within Cluster II and III, indicating the heterogeneity of UC mucosa.

### Three stable subtypes in UC were determined by CrossICC

**Fig. 3:**
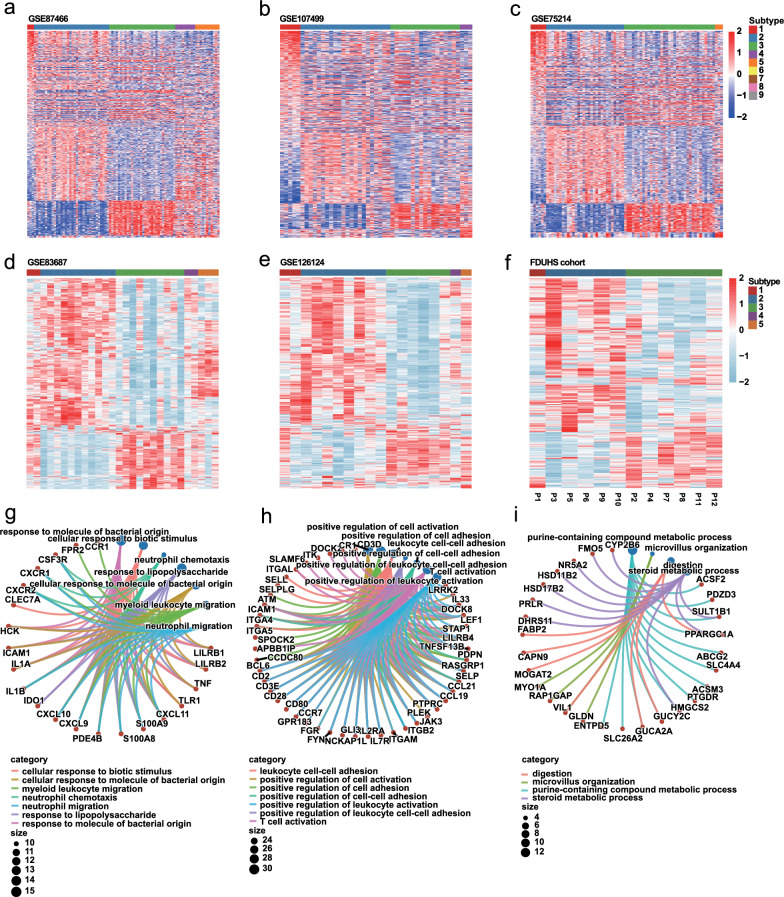
CrossICC assigned UC into three stable subtypes. **a**–**c** CrossICC identified subtypes in training cohorts **a** GSE87466, **b** GSE107499 and **c** GSE75214. **d**–**e** CrossICC validated the stability of subtypes in validation cohorts **d** GSE83687 and **e** GSE126124. **f** The application of CrossICC on a small FDUHS cohort quantified by RNA-seq. **g**–**i** GO analysis of the feature genes of **g** Subtype 1, **h** Subtype 2 and** i** Subtype 3

From the aforementioned data, the heterogeneity of UC mucosa at a transcription level had been initially identified. But as shown in WGCNA, the uniqueness of Cluster I has yet to be revealed, which may be attributed to the confounding effect accompanied by the removal of the batch effect. Thus, we leveraged CrossICC as the main subtyping strategy, which provided a novel pipeline that did not rely on eliminating batch effects. Given the 3 datasets, a variety of subtypes along with their marker genes were identified, in which Subtypes 1, 2, and 3 existed steadily in each dataset, while Subtypes 4 and 5 were unreliably missing in individual cohorts (Fig. 3a–c). Additionally, the detailed subtyping results and marker genes of each subtype were recorded (Additional file 1: Tables S6, S7).

Next, CrossICC was validated on two external datasets GSE83687 and GSE126124, with all the feature genes (n = 540) derived from CrossICC applied. It was confirmed that the Subtype 1, 2, and 3 could be distinguished in external cohorts (Fig. 3d, e). Furthermore, to demonstrate the clinical applicability of the subtypes, we imposed CrossICC on the RNA-seq results of 12 patients enrolled from Huashan Hospital, Fudan Univerisity (FDUHS). We discovered that CrossICC could also identify distinct subtypes in the FDUHS cohort, including the stable Subtypes 1, 2, and 3 (Fig. 3f, Table 1).

Moreover, Biological process enrichment for the exemplar genes in each subtype revealed that neutrophils were activated in Subtype 1 (Fig. 3g). Marker genes in Subtype 2 were involved in the activation pathways of multiple immune cells (Fig. 3h). Also, marker genes in Subtype 3 no longer participated in immune-related pathways (Fig. 3i). Taken together, we demonstrated that the 3 subtypes appear steady in UC patients with distinctive features.

### Three subtypes of UC exhibit heterogeneous patterns in immune cell infiltration

**Fig. 4:**
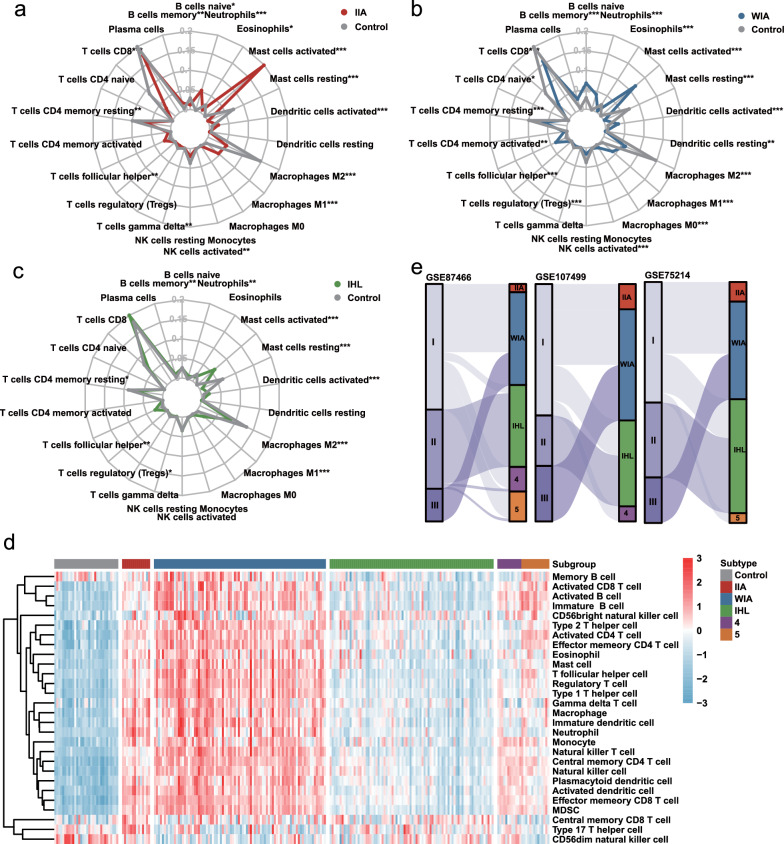
Immune cell infiltration of the 3 subtypes in CrossICC. **a**–**c** Immune cell infiltration proportion of **(a)** Subtype 1 (IIA), **b** Subtype 2 (WIA) and **c** Subtype 3 (IHL) by CIBERSORT. **d** ssGSEA evaluated the immune cell infiltration abundance in three subtypes. **e** Sankey Diagram of the correlation between consensus clustering and CrossICC

Considering that UC is a disease highly associated with the immune response, we wondered whether immune cell infiltration could recapitulate the difference among the 3 subtypes. CIBERSORT was applied to evaluate the infiltration proportion of different immune cells in each subtype of UC. A remarkable elevation of neutrophils and mast cells was observed in Subtype 1 compared to normal tissues, which was labeled as IIA UC (Fig. 4a). On the other hand, we noticed a fierce activation of multiple immune cells but a smaller proportion of mast cells in Subtype 2, which we labeled as WIA UC (Fig. 4b). Interestingly, the distribution of immune cells in Subtype 3 exhibited similarity with normal mucosa (Fig. 4c). Thus, Subtype 3 was termed IHL UC. Furthermore, the enrichment of 28 immune cell gene sets via ssGSEA robustly verified the different infiltration patterns in the 3 subtypes of UC (Fig. 4d).

Subsequently, to integrate the relationships among clusters identified by consensus clustering and the IIA, WIA and IHL of CrossICC, Sankey diagrams were plotted in each dataset. Notably, Cluster III was projected to IHL as expected, and Cluster II was stably projected to WIA. For Cluster I, projection to IIA, WIA, and IHL, as well as some undetermined subtypes were derived (Fig. 4e). Hence, the heterogeneity identified by consensus clustering could be demonstrated in CrossICC, which identified IHL to be a subtype with a normal-like immune microenvironment, while the clinical impact of IHL requires further exploration.

### IHL owns the best response to anti-TNF or anti-integrin therapy

**Fig. 5:**
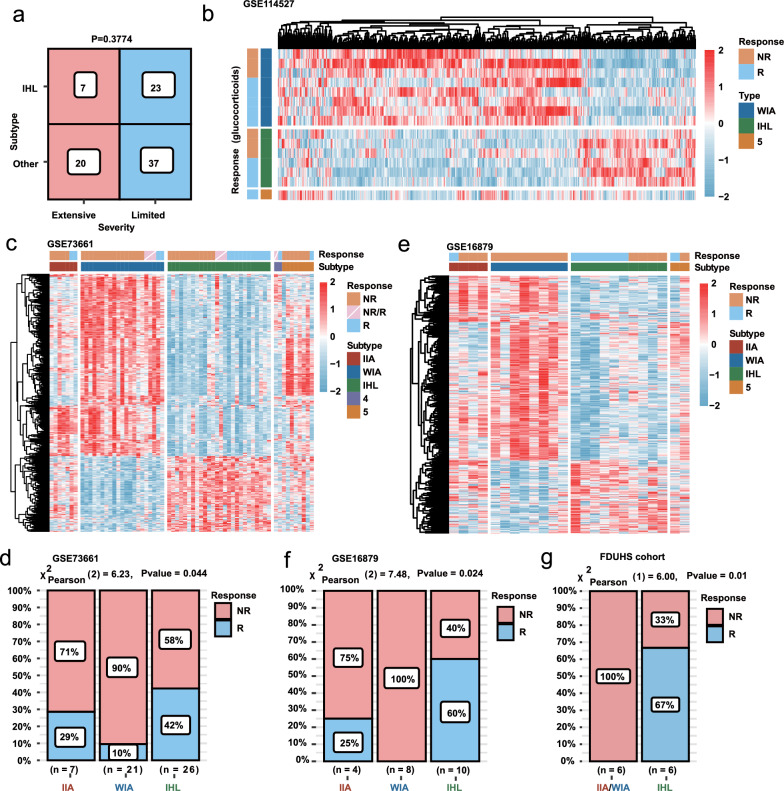
IHL owned the best response to anti-TNF or anti-integrin therapy **a** Severity of IHL under endoscopy was not statistically different from other subtypes. **b** There was no difference in the treatment of glucocorticoids for each subtype. **c**–**d** Three subtypes exhibited different response rate to Infliximab or Vedolizumab therapy in GSE73661. **e**–**f** Three subtypes exhibited different response rate to Infliximab therapy in GSE16879. **g** Three subtypes exhibited different response rate to Infliximab or Vedolizumab therapy in FDUHS cohort (IIA n = 1, WIA n = 5, IHL n = 6)

To ensure the subtyping of UC possesses clinical significance, we focused on the differences in the clinical outcomes and drug responses among subtypes, especially the IHL subtype and other immune-activated subtypes. First, we compared the disease severity between IHL and other subtypes. Interestingly, the distribution of limited and extensive diseases showed no significant differences between the two groups (*P* = 0.3774) (Fig. 5a), which indicated that the two groups cannot be differentiated by colonoscopy. In addition, with the subtype prediction of GSE114527, we also demonstrated that the response to glucocorticoids of IHL and other subtypes showed no significant difference (Fig. 5b).

Hence, with regard to the immune heterogeneity, GSE73661 and GSE16879, which contained the treatment outcomes of biological agents, were used to evaluate the response rate of IIA, WIA, and IHL subtypes to biological agents. Notably, Infliximab and Vedolizumab yielded the highest response rate in IHL (*P* = 0.044) (Fig. 5c, d). Besides, the enrichment of the key therapeutic targets further exhibited that multiple treatment targets were activated in IIA and WIA, which could explain the poor treatment response of these two subtypes (Additional file 1: Figure S4a). For GSE16879, it was also explicit that WIA showed the worst Infliximab treatment response rate (100% non-response), while the response rate of IHL was the best (*P* = 0.024) (Fig. 5e, f). Moreover, the enrichment of therapeutic targets also exhibited similar results (Additional file 1: Figure S4b). Remarkably, the patient who responded to biological agents in the FDUHS cohort also belonged to IHL, while IIA and WIA subtypes had no responders (Fig. 5g, Table 1, Additional file 1: Table S2). Additionally, 25–29% of IIA patients in GSE73661 and GSE16879 presented a drug response, which molecularly showed the activation of the tryptophan metabolic pathway but possessed the same immune patterns with IIA non-responders (Fig. 4c, d). Collectively, we demonstrated that UC patients with the IHL subtype should benefit from biological agents in clinical therapy.

### The ratio of PRLR to TNFSF13B predicts IHL accurately

**Fig. 6:**
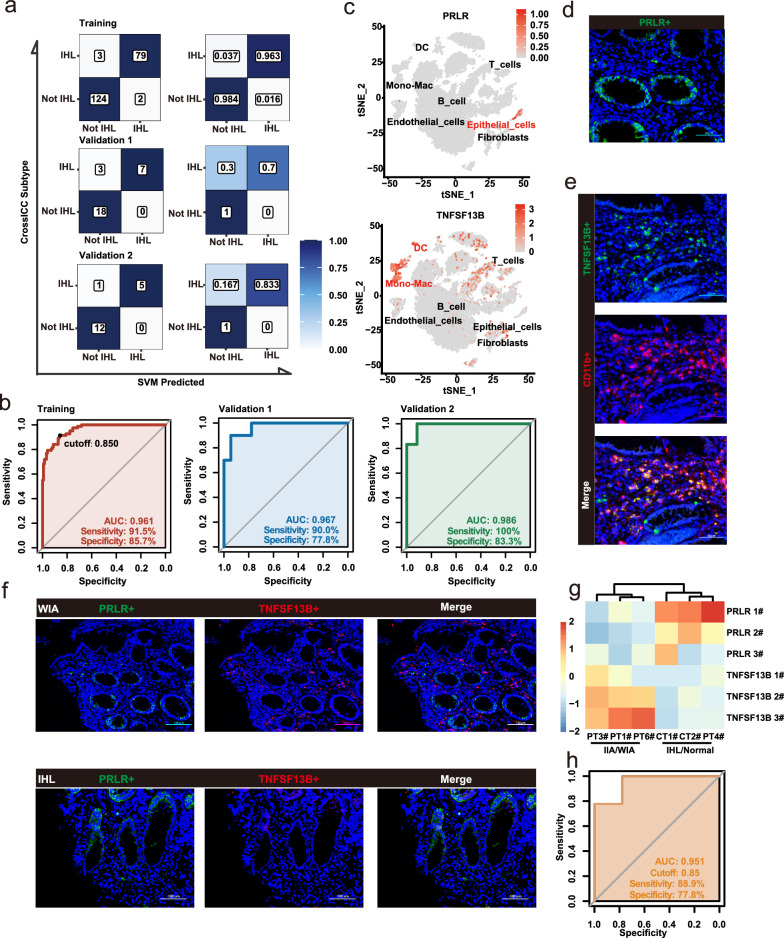
Clinically applicable panels and biomarkers for the subtyping. **a** Confusion matrixes and tables of true positive rate, false positive rate, true negative rate and false negative rate of the 16-gene SVM model. **b** Receiver operating characteristic curve evaluated the predictive potent of the ratio of PRLR and TNFSF13B and the sensitivity and specificity with the cutoff ratio set to 0.85. **c** Single-cell RNA sequencing (GSE182270) presented the location of PRLR and TNFSF13B. **d** Immunofluorescence confirmed the location of PRLR (Green: PRLR). **e** Immunofluorescence confirmed the location of TNFSF13B (Green: TNFSF13B, red: CD11b). **f** Immunofluorescence co-staining of PRLR and TNFSF13B in representative WIA (upper panel) and IHL subtype patients (lower panel). **g** Heatmap of immunofluorescence positivity signal rates of PRLR and TNFSF13B for patients (PT) and normal controls (CT). **h** Predictive ability of the ratio between immunofluorence signals of PRLR and TNFSF13B for determining IHL subtypes/normal and IIA/WIA subtypes

Since patients with the IHL subtype showed promise in benefiting from biological agent therapy, the identification of IHL patients to optimize the choice of treatment is needed. As shown in the workflow diagram (Fig. 1), random forest from the Boruta package was first applied to the 208 samples as a training cohort, filtering the total 460 non-repetitive marker genes to 108. Secondly, LASSO regression was applied to further reduce the variates to 16 (Table 2). Furthermore, the public scRNA-Seq dataset GSE182270 indicated the location of each of the 16 genes (Additional file 1: Figure S5a). Subsequently, SVM was built based on the scaled 16 genes, with the best gamma value set to 0.1 and cost set to 1. SVM exhibited an accuracy of 97.6% in the training cohort and reached an accuracy of 89.3% in the validation set GSE83687, as well as 94.4% in GSE126124 (Fig. 6a, left panel). The true positive rate, false positive rate, true negative rate, and false negative rate were also exhibited (Fig. 6a, right panel), which demonstrated that the 16 genes left were indeed important distinguishing factors.

**Table 2:** Sixteen genes and their coefficients obtained by Lasso regression

Gene	Coefficient
TLR1	−0.3344
C1orf162	−0.0740
TNFSF13B	−1.1931
CHI3L2	−0.7513
FAM126A	−0.5278
LAMP3	−0.0165
SERPINB9	−0.1704
FGR	−2.4344
SLC16A6	−0.8533
ELMO1	−0.2578
TFEC	−0.0731
PRLR	1.2814
NEDD4L	0.3394
SLC39A5	1.0169
PCSK6	0.9738
TRAF1	−0.8707

Considering that the detection of 16 genes is still cumbersome, we sought to determine the least number of genes that could provide a concise approach to identify the IHL subtype. Since the crucial issue was to find a uniform prediction threshold among samples from different batches, we predicted that the expression ratio of two genes might be an ideal predictive signature. First, PRLR, SLC39A5, FGR, and TNFSF13B were selected because their LASSO coefficient (coef) was greater than 1 or less than -1 (Table 1). Among the four genes, higher expression of PRLR or SLC39A5 represented a greater possibility of IHL (LASSO coef > 1). Conversely, higher expression of TNFSF13B and FGR represented a smaller possibility of IHL (LASSO coef < −1). Different ratio combinations of two genes with coefficients greater than 1 and less than −1 were tested, respectively, including PRLR/FGR, PRLR/TNFSF13B, SLC39A5/FGR, and SLC39A5/TNFSF13B. Among the 4 combinations, only PRLR/TNFSF13B showed comparable predictive performance to the 16-gene SVM model in both the training and validation sets (training cohort AUC = 0.961, validation cohort GSE83687 AUC = 0.967 and GSE126124 AUC = 0.986) (Additional file 1: Table S8, Fig. 6b). With the best cutoff ratio set to 0.85 obtained from the training set, the sensitivity and specificity of the two-gene ratio was also exhibited, which indicated that the ratio of PRLR/TNFSF13B might be a powerful index to determine the subtype (Fig. 6b).

To investigate the potential mechanism for how PRLR and TNFSF13B became reliable markers for subtyping, we referred to the scRNA-seq data. The data suggested that PRLR was expressed by epithelial cells, while TNFSF13B was mainly expressed in monocyte-derived myeloid cells, especially macrophages in both datasets GSE182270 and GSE150115 (Fig. 6c, Additional file 1: Figure S5b, c). Immunofluorescence analysis also showed the location of the two genes. (Fig. 6d, e). Furthermore, GSE150115 demonstrated that TNFSF13B was indeed expressed in macrophages with a highly heterogeneous pattern in UC patients (Additional file 1: Figure S5d). To illustrate the clinical usability of PRLR to TNFSF13B ratio, we applied pathological sections from FDUHS cohort, containing IIA, WIA, IHL subtypes of UC patients and 2 normal colonic mucosa specimens. We co-stained PRLR and TNFSF13B and observed using immunofluorescence (Fig. 6f, upper panel: WIA, lower panel: IHL). Then, the positive signal rates of the two genes were counted under three random fields of view per section and obtained a total of 18 calculated results, which showed a contradicting trend between the IHL and IIA types between IHL/normal and IIA/WIA subtypes (Additional file 1: Table S9, Fig. 6g). Critically, co-stained immunofluorescence-based PRLR and TNFSF13B ratios could still effectively identify IHL subtype at the aforementioned cut-off value of 0.85 (AUC = 0.951, Sensitivity = 88.9%, Specificity = 77.8%) (Fig. 6h). Thus, there might be an interaction between PRLR in epithelial cells and TNFSF13B in macrophages, which can affect the immune status and treatment outcome of UC patients.

### Epithelial PRLR inhibited TNFSF13B through CXCL1-NF-κB signaling in macrophages

**Fig. 7:**
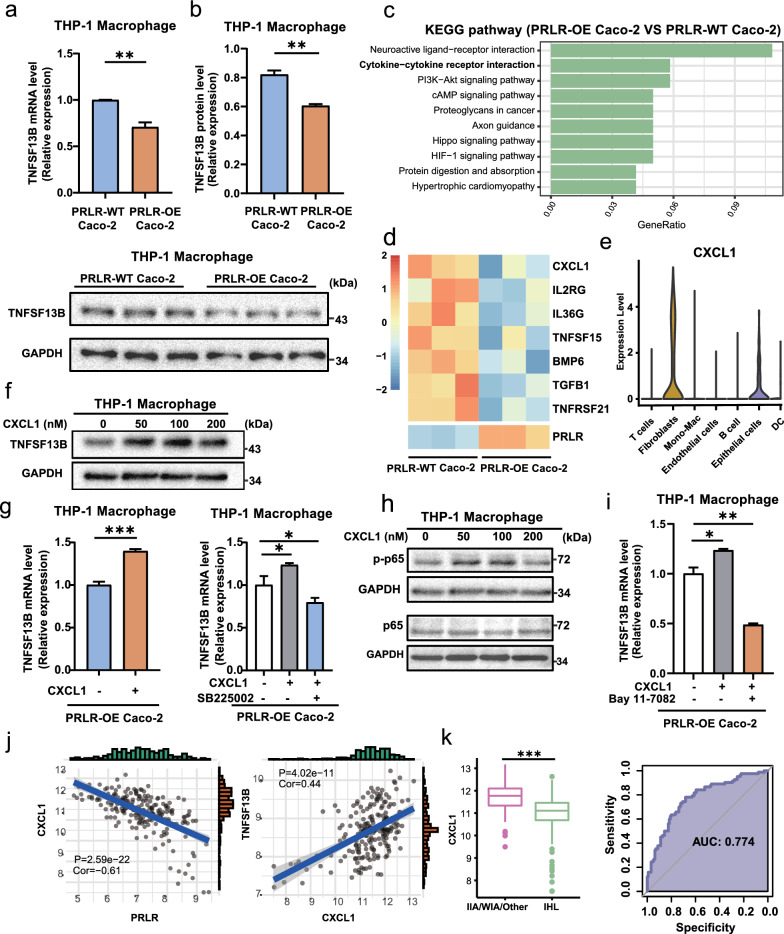
Epithelial PRLR inhibited TNFSF13B of macrophages through attenuated CXCL1-NF-κB signaling. THP-1 cells were pretreated with PMA for 48 h to induce the differentiation of macrophage. Then, these cells were stimulated with cultural supernatant of PRLR-overexpressed (OE) Caco-2 or PRLR-wide type (WT) Caco-2 for 24 h. **a**, **b** The mRNA and protein levels of TNFSF13B were detected by qRT-PCR and western blot, respectively**. c** RNA-sequencing was performed for the PRLR-WT (n = 3) and PRLR-OE (n = 3) Caco-2 cells after LPS stimulation for 48 h. KEGG enrichment for the significant down-regulated DEGs (Log2(Fold change) < -0.2, *P* value < 0.05) of PRLR-OE Caco-2 compared with PRLR-WT Caco-2 cells. **d** Heatmap of the significantly down-regulated genes in cytokine-cytokine interaction pathway. **e** scRNA-seq from GSE182270 exhibited the expression levels of CXCL1 in different cell types. **f**, **h** THP-1-derived macrophages were stimulated with the supernatant of PRLR-OE Caco-2, and supplemented with different doses of CXCL1 for 24 h. TNFSF13B levels and phosphorylated NF-κB p65 were detected by western blot.** g**, **i** CXCR2 inhibitory SB225002 (10 μM) and NF-KB pathway inhibitor Bay 11–7082 (1 μM) were used to block the CXCL1-induced effect. The TNFSF13B levels were detected by qRT-PCR. **j** The correlation of PRLR or TNFSF13B with CXCL1 expression in the UC tissues (n = 208). **k** The CXCL1 expression in IIA/WIA and IHL, and the sensitivity of CXCL1 expression in IHL to distinguish from IIA/WIA

Finally, we sought to explore the interaction between PRLR in epithelial cells and TNFSF13B in macrophages, and uncover the underlying reason why the ratio of PRLR/TNFSF13B could identify the molecular sub-classification and predict the outcome of UC patients. First of all, Caco-2 cells, divided into overexpressed or wild-type PRLR (PRLR-OE or PRLR-WT, Additional file 1: Figure S6a), were stimulated with 1 μg/mL of LPS for 48 h respectively. Then, the cultural supernatant of these Caco-2 cells was collected to stimulate PMA-pretreated THP-1 cells (Fig. 1), which was widely used as human macrophages [28]. As expected, TNFSF13B expression in THP-1 cells upon PRLR-OE Caco-2-derived cultural supernatant was much lower than that of PRLR-WT Caco-2-derived cultural supernatant, verified in both mRNA and protein levels (Fig. 7a, b). Results indicated that PRLR in epithelial cells was negatively correlated with TNFSF13B in macrophages. Meanwhile, we performed RNA-sequencing for the PRLR-WT (n = 3) and PRLR-OE (n = 3) Caco-2 cells after LPS stimulation for 48 h. Interestingly, KEGG enrichment for the significantly down-regulated DEGs (Log2(Fold change) < −0.2, *P* < 0.05) revealed that cytokine-cytokine receptor interaction was downregulated in the PRLR-OE Caco-2 group compared with that of the PRLR-WT Caco-2 group (Fig. 7c). Among the down-regulated genes participating in cytokine-cytokine receptor interaction pathways, CXCL1 was selected for the largest fold change when comparing the PRLR-OE Caco-2 group with the PRLR-WT Caco-2 group (Fig. 7d). Also, scRNA-seq from GSE182270 offered evidence that epithelial cells were one of the two main sources of CXCL1, while other cytokines were rarely expressed in epithelial cells (Fig. 7e, Additional file 1: Figure S6b). Consistently, an additional supplement of CXCL1, especially at 100 nM, would recover the expression level of TNFSF13B in THP-1 cells stimulated by the supernatant of PRLR-OE Caco-2 (Fig. 7f). Furthermore, the above phenomenon could be abolished using CXCR2 inhibitory SB225002 (Fig. 7g), indicating that the PRLR-mediated TNFSF13B down-regulation in THP-1 cells depended on the decreased secretion of CXCL1 in Caco-2 cells. Since previous studies showed that CXCL1 could activate NF-κB signaling [29], we detected the NF-κB signaling in CXCL1-treated THP-1 cells. Compared with THP-1 cells without CXCL1 back-complementation, the phosphorylated NF-κB p65 was elevated in CXCL1-treated THP-1 cells (Fig. 7h). As expected, the CXCL1-induced TNFSF13B up-regulation was abrogated by the NF-KB inhibitor Bay 11–7082 (Fig. 7i). Alternations of protein levels of TNFSF13B and NF-κB p65 after the use of SB225002 and Bay 11–7082 were also confirmed (Additional file 1: Figure S6c). Lastly, we observed a negative correlation between PRLR and CXCL1 expression, but a positive correlation between CXCL1 and TNFSF13B expression in a cohort of UC patients (Fig. 7j). Moreover, IIA/WIA patients owned higher expression of CXCL1 than IHL, and CXCL1 had a predictive value in distinguishing IIA/WIA and IHL (AUC = 0.774), implying the bridge role of CXCL1 (Fig. 7k). Taken together, PRLR-mediated the decreased secretion of CXCL1 in epithelial cells suppresses TNFSF13B expression through CXCR2-NF-KB pathway in macrophages, which subsequently attenuates inflammation. This may provide a rational explanation for why the ratio of PRLR/TNFSF13B can act as a reliable prediction for the molecular sub-classification and outcome of UC patients.

## Discussion

UC has imposed an increasing financial burden on the global healthcare system. The adverse effects caused by long-term treatment have received increasing attention [30, 31]. Thus, understanding the heterogeneity of UC to construct individualized treatment is urgently needed. In this study, based on 357 UC colon samples obtained from datasets, we first identified heterogeneity of UC with consensus clustering and WGCNA. Then, CrossICC classified the UC patients into three stable subtypes termed IIA, WIA, and IHL. Notably, IHL exhibited a normal intestinal mucosa-like immune cell infiltration pattern and presented the best response towards biological agents such as Infliximab and Vedolizumab, while WIA subtype had the worst response. Additionally, we demonstrated that the ratio of PRLR to TNFSF13B could be an effective biomarker to facilitate subtype screening for precise treatment in UC. The underlying mechanism may be that PRLR impaired CXCL1 secretion in epithelial cells, leading to the blockade of CXCR2-NF-κB-TNFSF13B pathway in macrophages.

Notably, among our newly-identified three UC subtypes, the main differences were the type and number of infiltrated immune cells, which have not been a point of interest in a previous study on CD patients, although the presence of molecular subtypes has been mentioned [8]. In the IIA subtype of UC, the most prominent characteristic is the elevation of the innate immune response, especially neutrophils. Studies have revealed that neutrophils in UC patients (not in CD patients) formed neutrophil extracellular traps (NETs) [32], which strengthened the production of TNF-α and IL-1β in mononuclear cells and the expression of DDIT4/REDD1 protein, leading to epithelial damage [32, 33]. In addition, the neutrophil-to-lymphocyte ratio was proposed to be a protective predictor of clinical relapse of UC [33], which showed concordance with the treatment outcome comparison in our study (Fig. 5). On the other hand, WIA identified in the study was characterized by the activation of both innate and adaptive immune, especially B cells and T follicular helper cells (Tfh). Tfh cells which express CXCR5, ICOS, and PD-1 are crucial for the stimulation of B cells [34]. Activated B cells secrete antibodies of high affinity and cytokines to exacerbate UC [35, 36]. Additionally, the elevation of activated dendritic cells was observed in WIA, which was regarded as a crucial cell in the crosstalk of innate and adaptive immune in UC [37, 38].

Intriguingly, we discovered that the IHL subtype of UC exhibited a normal-like immune landscape and the best response rate towards biological agents, while the IIA, especially WIA subtypes showed poor response rates. These data suggested that the over-activation of innate as well as adaptive immune both contributed to the resistance towards Infliximab and Vedolizumab. Similarly, recent studies revealed that CD patients with high infiltration of IgG plasma cells, inflammatory monocytes, T cells, and stromal cells exhibited resistance toward anti-TNF therapy [39]. So far, few studies elucidated the impact of the immune microenvironment on therapeutic outcomes in UC patients. Thus, one possible explanation for the limited efficacy of biological agents in clinical practice is that single pathway-target therapy tends to be insufficient to handle multiple activated immunopathological signaling [39, 40]. In addition, nearly a quarter of patients with IIA might experience remission of biologic agents, not due to alterations in immune cells, but possibly due to activation of specific anti-metabolic pathways such as tryptophan metabolism [41–43]. However, the sample size (n = 3) of IIA responders is currently not sufficient to build predictive models to identify these patients. Therefore, more large cohorts containing treatment outcomes are needed in the future to develop effective panels to predict the likely response of IIA patients.

Genes left by LASSO regression facilitate the understanding of the mechanisms of the heterogeneity of the immune microenvironment. In general, we noticed that the 16 genes reflected the balance between the inhibitory effect of epithelium and the activation of myeloid cells. TNFSF13B, also named as the B-cell-activating factor of the TNF family [44, 45], was considered a negative factor for the anti-TNF or anti-integrin therapy in this study, reflected by a significant upregulation in the WIA and IIA subtypes and a large LASSO coefficient (Table 1). Integrating scRNA-seq and Immunofluorescence co-localization, our study showed that TNFSF13B was predominantly expressed in monocyte-derived myeloid cells, especially macrophages, which might be the crucial immunopathological factors in UC. Interestingly, it was reported that there was a stratification of high and low plasma TNFSF13B in melanoma patients treated with anti-PD1/PDL1 therapy, which again hinted that TNFSF13B shared extensive heterogeneity between patients [46]. As a TNF ligand superfamily, TNFSF13B cannot be inhibited by conventional anti-TNFα agents such as infliximab [47]. Hence, we speculate that anti-TNF therapy only targets the TNF signaling, while the impact of bypass and associated factors cannot be suppressed, leading to drug resistance. Also, the TNF ligand superfamily and TNF receptor-associated factor might be potential targets for future treatment for the WIA or IIA subtypes [48–50].

Next, we attempted to elucidate the reason why patients (IHL) with higher expression of PRLR tend to be more sensitive to biological agents. Previous studies have demonstrated that prolactin could act on short-form PRLR to impede the TRAF-dependent innate immune response induced by IL-1β, which also restricts the signaling of TNF-α and TLR4 [51]. Anti-PRLR could recruit T cells and promote IFN-γ and TNF-α produced by Th1 cells, indicating that PRLR expressed in epithelial cells can suppress T cells [52]. Moreover, prolactin can also upregulate the expression of IL-10 and downregulate IL-1β, TNF-α, and IL-12 to suppress the inflammatory monocytes [53]. In this study, we suggested the novel role of PRLR expressed in intestinal epithelial cells, which inhibited the CXCL1-NF-κB-TNFSF13B pathway to attenuate inflammation. As a classical chemokine, CXCL1 has been reported to be up-regulated in the inflammatory mucosa of UC [54, 55]. However, the potential role of epithelial cell-derived CXCL1 in response to biological agents in UC has not been reported. Currently, only a few existing drugs targeting the CXCL1-CXCR2 interaction between epithelial cells and macrophages are available. The present study provided insight to the interplay between immune subtypes and inflammatory pathways in UC treatment.

Furthermore, our work emphasized the importance of human hormones and their receptors for immunophenotyping. Patients’ endogenous hormone and receptor levels have a great impact on immune homeostasis, which was partially revealed by previous studies [56, 57]. For instance, the estrogen receptor (ER) has been of critical value for subtyping in the field of breast cancer, and androgen receptors (AR) also suppressed CD8 + T cells function [58, 59]. We discovered that the prolactin receptor has the potential to inhibit TNF-associated factors as well as multiple cytokine-related pathways, while anti-TNF or anti-integrin therapy can have a synergistic effect with PRLR. Thus, the future of biological therapy for UC should focus more on monitoring the patient's endogenous hormones and their receptors, leveraging them for immunomodulation.

## Conclusion

Collectively, our study revealed the molecular and immune heterogeneity of UC and classified patients into three subtypes: IIA, WIA, and IHL. In addition, we established a concise subtyping approach and explored the potential immunomodulatory mechanisms, which may facilitate precision medicine for UC patients.

## Supplementary Information

**Additional file 1: Figure S1.** Principal component analysis (PCA) demonstrated the overview and selection of samples. (a) PCA exhibited the separation of normal (n = 21) and diseased (n = 87) tissues and the mixing of limited (n = 60) and extensive (n = 27) tissues in GSE87466. (b) PCA exhibited separation of lesional (n = 47) and non-lesional (n = 40) tissues in GSE107499 (c) PCA exhibited the separation of active (n = 74), normal (n = 11) and inactive (n = 23) tissues in GSE75214 (d) PCA of the 3 datasets before removing batch effect. (e–f) PCA of the 3 datasets after removing batch effect, annotated by (e) data resources and (f) types of mucosal lesions (UC included = 208). **Figure S2.** Consensus score of different clustering numbers. **Figure S3.** WGCNA of the samples. (a) Three samples were removed for the outliers. (b) Power selection based on the R^2. (c) Clusters of the gene module. (d) Detailed GO enrichment result for each cluster. **Figure S4.** Enrichment of the key therapeutic targets. (a) Enrichment of the key therapeutic targets in GSE73661. (b) Enrichment of the key therapeutic targets in GSE16879. (c) Immune patterns comparison between IIA non-responders (IIA-NR) and IIA responders (IIA-R). (d) GO enrichment of genes upregulated in IIA responders (Log2FC > 1, Pvalue < 0.05). **Figure S5.** scRNA-sequencing of GSE182270 and GSE150115. (a) The localization of 16 genes in GSE182270. (b) The UMAP plot of the cells in GSE150115. (c) The localization of TNFSF13B and PRLR in GSE150115. (d) The expression of TNFSF13B on monocytes derived from different patients. **Figure S6.** Epithelial PRLR inhibited TNFSF13B of macrophages through attenuated CXCL1-NF-κB signaling (a) PRLR-overexpressed Caco-2 cells were constructed. (b) Cellular localization of differentially expressed cytokine related genes (c) CXCR2 inhibitory SB225002 (10 μM) and NF-KB pathway inhibitor Bay 11–7082 (1 μM) were used to block the CXCL1-induced effect. The TNFSF13B levels were detected by Western blot. **Table S1.** Detailed information of the included datasets. **Table S2.** Detailed Mayo scores of enrolled patients at baseline and post-treatment. **Table S3.** Included therapeutic pathway genes and their source databases. **Table S4.** Comparison of clinical traits between 3 clusters. **Table S5.** GO enrichment for each WGCNA module. **Table S6.** Marker genes of each CrossICC subtype. **Table S7.** The CrossICC subtypes of each sample. **Table S8.** The performance of different combinations of two-gene ratio. **Table S9.** Immunofluorescence signal positive rates of PRLR and TNFSF13B and ratios.

## Data Availability

Public data is available in a GEO database (https://www.ncbi.nlm.nih.gov/geo/) and RNA-Sequencing data in the study was uploaded into GEO database with the number GSE220815.
