# Supplementary material for: A precise molecular subtyping of ulcerative colitis reveals the immune heterogeneity and predicts clinical drug responses
Source: J Transl Med. 2023 Jul 13;21:466. doi: 10.1186/s12967-023-04326-w (PMC10347743; doi:10.1186/s12967-023-04326-w)
Supplement: Supplementary file 1 — Additional file 1: Figure S1. Principal component analysis (PCA) demonstrated the overview and selection of samples. (a) PCA exhibited the separation of normal (n = 21) and diseased (n = 87) tissues and the mixing of limited (n = 60) and extensive (n = 27) tissues in GSE87466. (b) PCA exhibited separation of lesional (n = 47) and non-lesional (n = 40) tissues in GSE107499 (c) PCA exhibited the separation of active (n = 74), normal (n = 11) and inactive (n = 23) tissues in GSE75214 (d) PCA of the 3 datasets before removing batch effect. (e–f) PCA of the 3 datasets after removing batch effect, annotated by (e) data resources and (f) types of mucosal lesions (UC included = 208). Figure S2. Consensus score of different clustering numbers. Figure S3. WGCNA of the samples. (a) Three samples were removed for the outliers. (b) Power selection based on the R^2. (c) Clusters of the gene module. (d) Detailed GO enrichment result for each cluster. Figure S4. Enrichment of the key therapeutic targets. (a) Enrichment of the key therapeutic targets in GSE73661. (b) Enrichment of the key therapeutic targets in GSE16879. (c) Immune patterns comparison between IIA non-responders (IIA-NR) and IIA responders (IIA-R). (d) GO enrichment of genes upregulated in IIA responders (Log2FC > 1, Pvalue < 0.05). Figure S5. scRNA-sequencing of GSE182270 and GSE150115. (a) The localization of 16 genes in GSE182270. (b) The UMAP plot of the cells in GSE150115. (c) The localization of TNFSF13B and PRLR in GSE150115. (d) The expression of TNFSF13B on monocytes derived from different patients. Figure S6. Epithelial PRLR inhibited TNFSF13B of macrophages through attenuated CXCL1-NF-κB signaling (a) PRLR-overexpressed Caco-2 cells were constructed. (b) Cellular localization of differentially expressed cytokine related genes (c) CXCR2 inhibitory SB225002 (10 μM) and NF-KB pathway inhibitor Bay 11–7082 (1 μM) were used to block the CXCL1-induced effect. The TNFSF13B levels were detected by Western blot. Ta [file 12967_2023_4326_MOESM1_ESM.docx]

**Supplementary Data**

**A Precise Molecular Subtyping of Ulcerative Colitis Reveals the Immune Heterogeneity and Predicts Clinical Drug Responses**

Shaocong Mo^1,2#^, Bryan Jin^1#^, Yujen Tseng^1#^, Lingxi Lin^1^, Lishuang Lin^1,3^, Xin Shen^1^, Huan Song^1^, Mingjia Kong^1^, Zhongguang Luo^1^, Yiwei Chu^4^, Chen Jiang^5^, Zhiwei Cao^6,7^, Jie Liu^1,2*^, Feifei Luo^1,2*^

^1^ Department of Digestive Diseases, Huashan Hospital, Fudan University, Shanghai 200040, China

^2^ National Clinical Research Center for Aging and Medicine, Huashan Hospital, Fudan University, Shanghai 200040, China

^3^ Department of Pathology, Huashan Hospital, Fudan University, Shanghai, China.

^4^ Biotherapy Research Center, Department of Immunology, School of Basic Medical Sciences and Institute of Biomedical Sciences, Fudan University, Shanghai 200032, China

^5^ Department of Pharmaceutics, School of Pharmacy, Fudan University, Shanghai 201203, China

^6^ School of Life Sciences, Fudan University, Shanghai 200433, China.

^7^ School of Life Sciences and Technology, Tongji University, Shanghai 200092, China.

^#^ These authors contributed equally to this work.

^*^ To whom correspondence should be addressed:

Dr. Feifei Luo, Tel/Fax: +86-21-54237769, E-mail: [feifeiluo@fudan.edu.cn](mailto:feifeiluo@fudan.edu.cn), or Dr. Jie Liu, Tel/Fax: +86-21-52888236, E-mail: [jieliu@fudan.edu.cn](mailto:jieliu@fudan.edu.cn); Department of Digestive Diseases, Huashan Hospital, Fudan University, 12 Wulumuqi Middle Road, Shanghai 200040, China.

**Disclaimers:** the authors declare no conflict of interest.

**Source(s) of support:** This study was supported by the National Natural Science Foundation of China (82121002, 81870375, 81870456).

**Word count:** Abstract 251 words, Text 5503 words

**Number of figures:** 7

**Number of tables:** 2

**Disclosure of relationships and activities:** All authors uphold the integrity of the work, approved the manuscript in its entirety, and are accountable for all aspects of the work.

**Supplementary Figures**


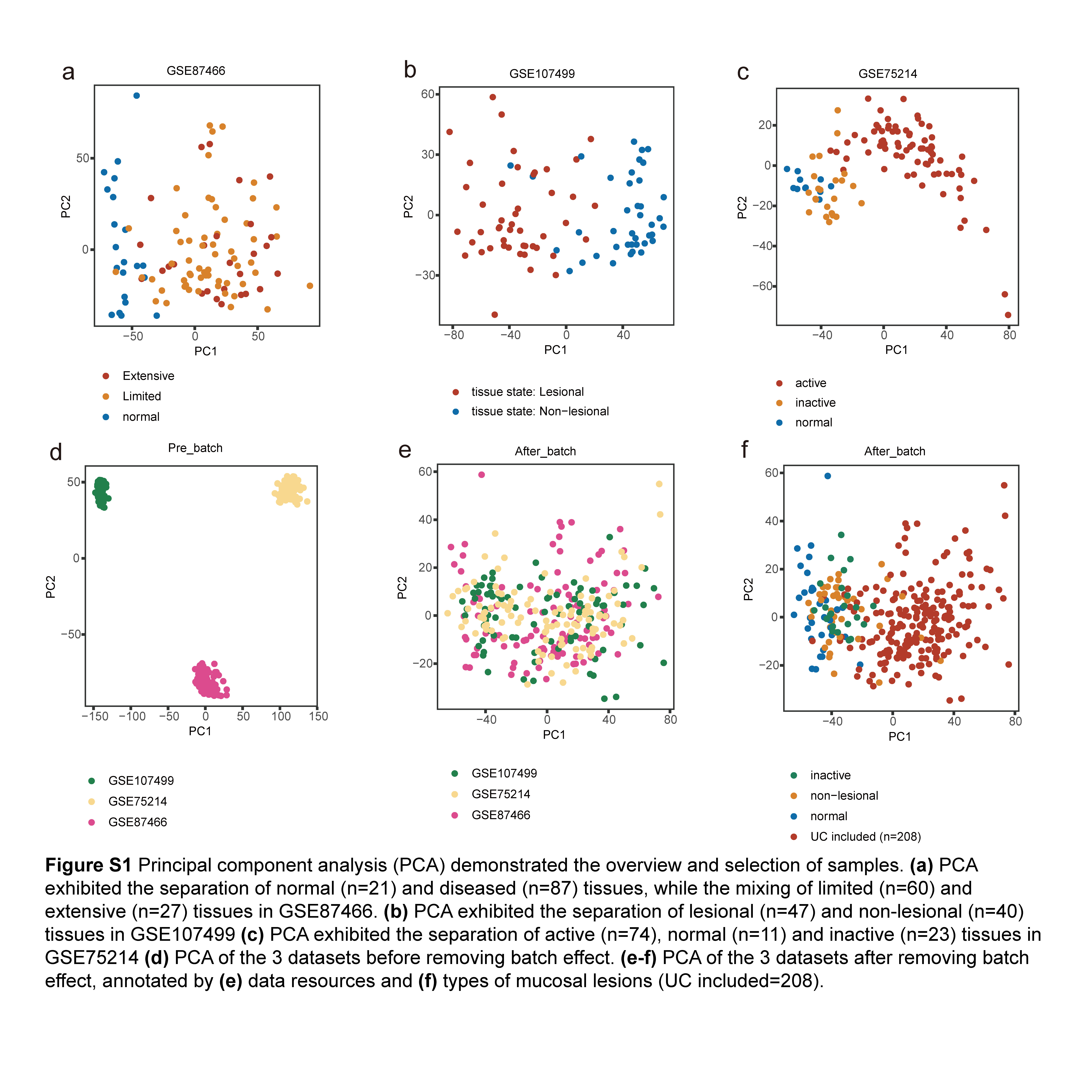


**Figure S1.** Principal component analysis (PCA) demonstrated the overview and selection of samples. (**a**) PCA exhibited the separation of normal (n=21) and diseased (n=87) tissues and the mixing of limited (n=60) and extensive (n=27) tissues in GSE87466. (**b**) PCA exhibited separation of lesional (n=47) and non-lesional (n=40) tissues in GSE107499 (**c**) PCA exhibited the separation of active (n=74), normal (n=11) and inactive (n=23) tissues in GSE75214 (**d**) PCA of the 3 datasets before removing batch effect. (**e-f**) PCA of the 3 datasets after removing batch effect, annotated by (e) data resources and (f) types of mucosal lesions (UC included=208).


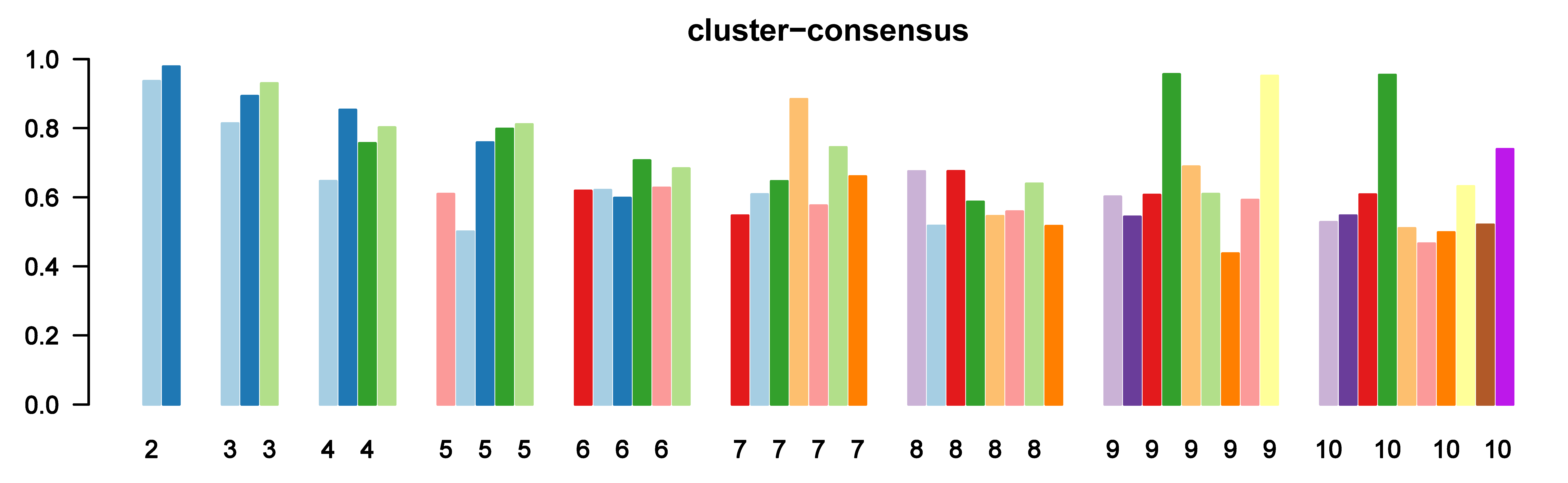


**Figure S2.** Consensus score of different clustering numbers.





**Figure S3.** WGCNA of the samples. (**a**) Three samples were removed for the outliers. (**b**) Power selection based on the R^2. (**c**) Clusters of the gene module. (**d**) Detailed GO enrichment result for each cluster.


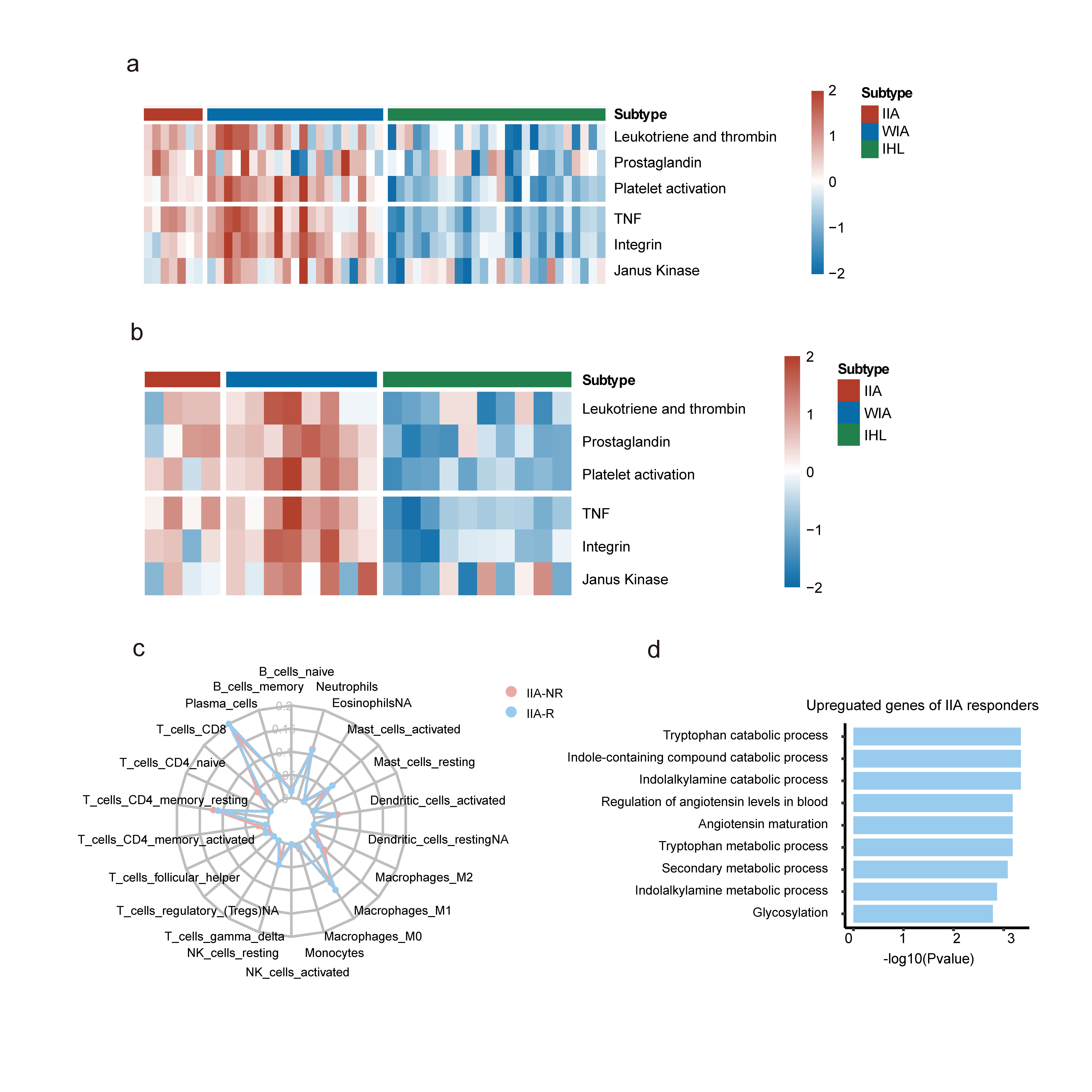


**Figure S4.** Enrichment of the key therapeutic targets. (**a**) Enrichment of the key therapeutic targets in GSE73661. (**b**) Enrichment of the key therapeutic targets in GSE16879. (**c**) Immune patterns comparison between IIA non-responders (IIA-NR) and IIA responders (IIA-R). (**d**) GO enrichment of genes upregulated in IIA responders (Log2FC>1, Pvalue<0.05).


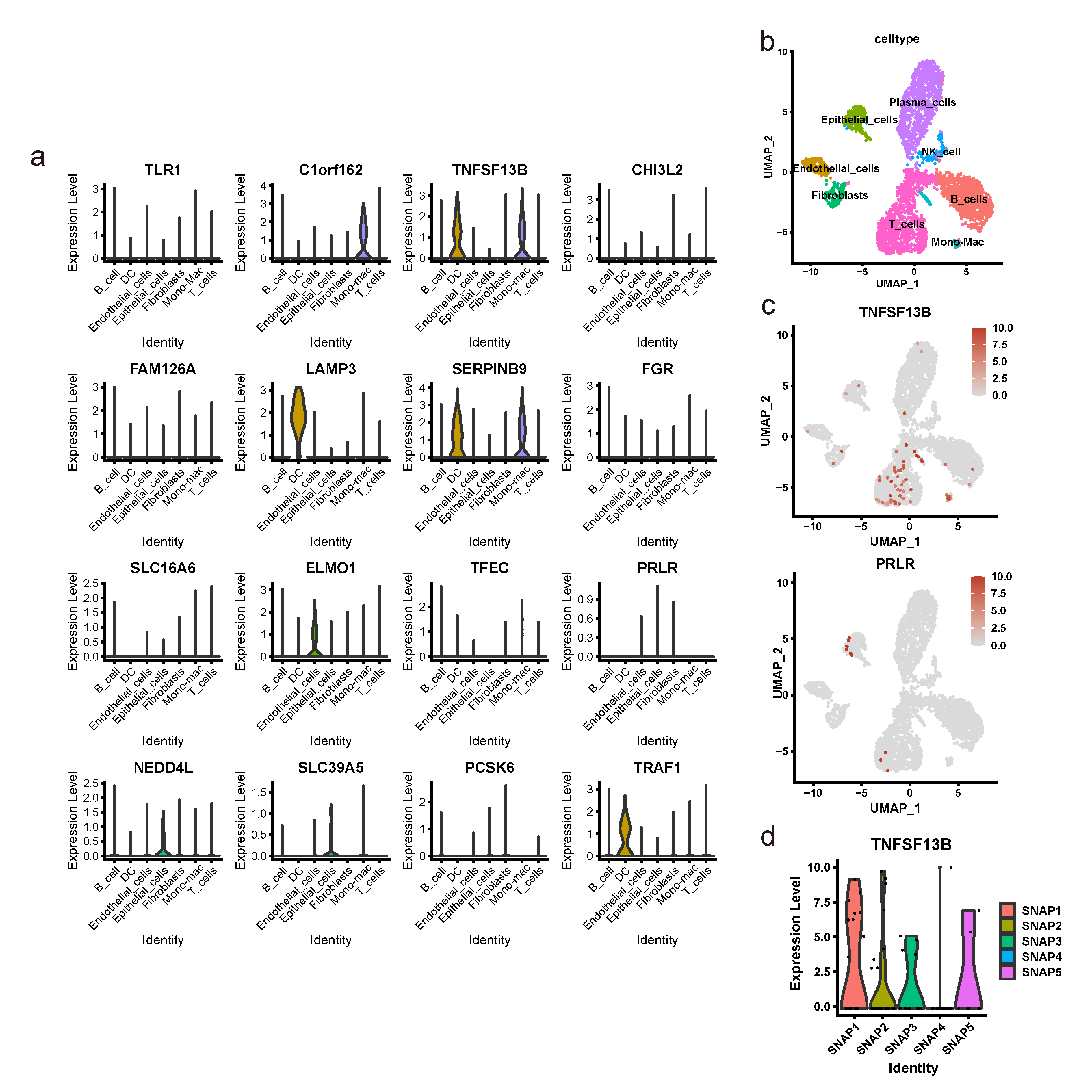


**Figure S5.** scRNA-sequencing of GSE182270 and GSE150115. (**a**) The localization of 16 genes in GSE182270. (**b**) The UMAP plot of the cells in GSE150115. (**c**) The localization of TNFSF13B and PRLR in GSE150115. (**d**) The expression of TNFSF13B on monocytes derived from different patients.


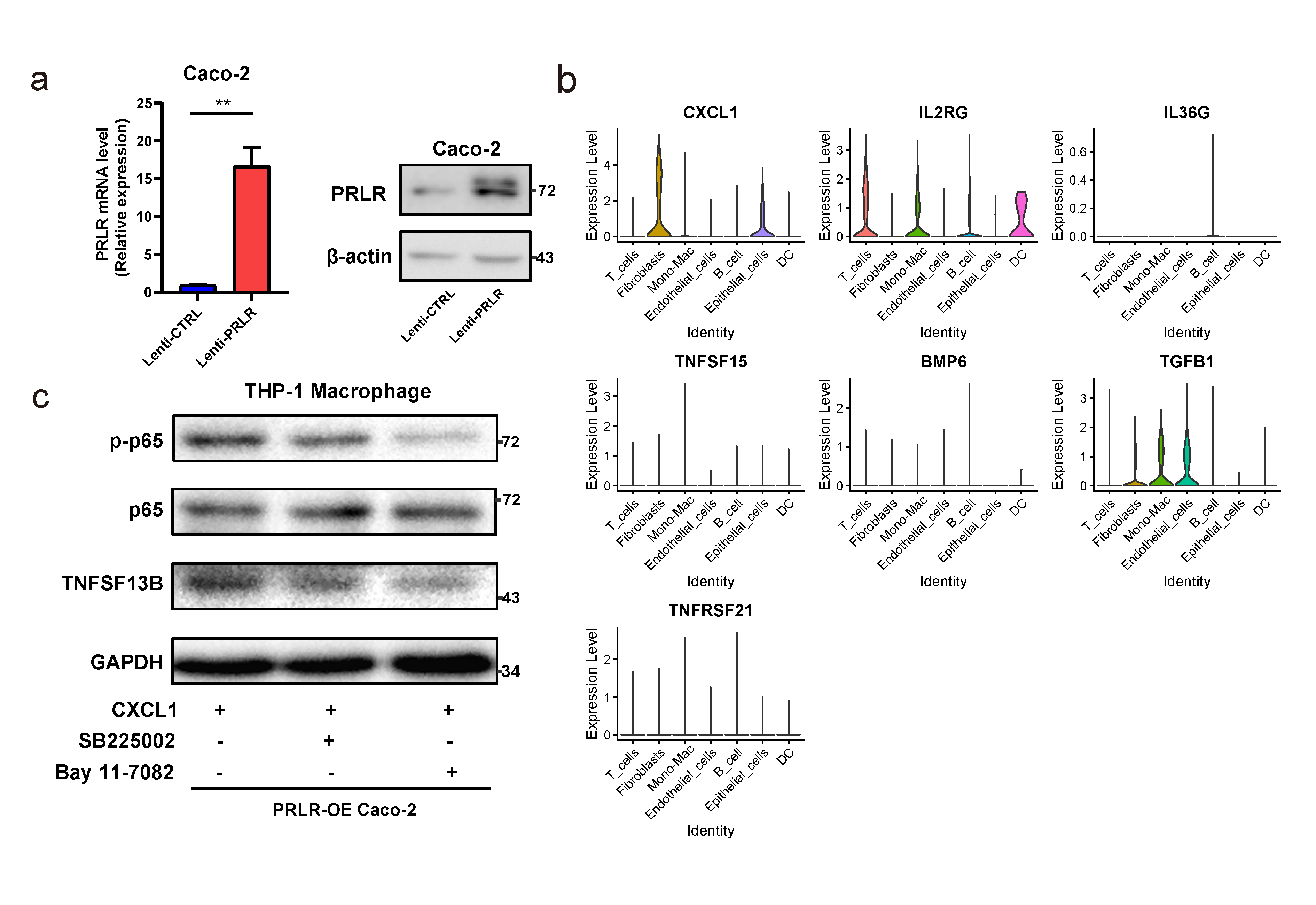


**Figure S6.** Epithelial PRLR inhibited TNFSF13B of macrophages through attenuated CXCL1-NF-κB signaling (**a**) PRLR-overexpressed Caco-2 cells were constructed. (**b**) Cellular localization of differentially expressed cytokine related genes (**c**) CXCR2 inhibitory SB225002 (10 μM) and NF-KB pathway inhibitor Bay 11-7082 (1 μM) were used to block the CXCL1-induced effect. The TNFSF13B levels were detected by Western blot.

**Supplementary Tables**

**Table S1.** Detailed information of the included datasets

| **Datasets** | **Description** | **Contributor**  **[Reference Number]** | **Citation (Pubmed ID)** | **Platform** | **UC Sample size** | **application** |
| --- | --- | --- | --- | --- | --- | --- |
| GSE87466 | Gene expression profiling of mucosal biopsies from adult patients with moderately to severely active ulcerative colitis | Li K et al. [9] | 29401083 | GPL13158 Affymetrix HT HG-U133+ PM Array Plate | 87 | Training |
| GSE107499 | Expression data from Ulcerative Colitis subjects | Gordon WM |  | GPL15207 Affymetrix Human Gene Expression Array | 47 |  |
| GSE75214 | Mucosal gene expression profiling in patients with inflammatory bowel disease study | Arijs I et al. [10] | 28885228 | GPL6244 Affymetrix Human Gene 1.0 ST Array | 74 |  |
| GSE83687 | A functional genomics predictive network model identifies regulators of inflammatory bowel disease: Mount Sinai Hospital (MSH) Population Specimen Collection and Profiling of Inflammatory Bowel Disease | Peters LA et al. [11] | 28892060 | GPL16791 Illumina HiSeq 2500 | 28 | Validation |
| GSE126124 | Concordance between gene expression in peripheral whole blood and colonic tissue in children with inflammatory bowel disease. | Palmer NP et al. [12] | 31618209 | GPL6244 Affymetrix Human Gene 1.0 ST Array | 18 |  |
| GSE114527 | A systems biology approach identifies ANP32E and other biomarkers of glucocorticoid refractoriness in ulcerative colitis | Lorén V et al. [13] | 30329026 | GPL14951 Illumina HumanHT-12 WG-DASL V4.0 R2 expression beadchip | 15 | Drug response comparison |
| GSE73661 | The effect of vedolizumab (anti-α4β7-integrin) therapy on colonic mucosal gene expression in patients with ulcerative colitis (UC) | Arijs I et al. [14] | 27802155 | GPL6244 Affymetrix Human Gene 1.0 ST Array | 64 |  |
| GSE16879 | Mucosal expression profiling in patients with inflammatory bowel disease before and after first infliximab treatment | Arijs I et al. [15] | 19956723 | GPL570 Affymetrix Human Genome U133 Plus 2.0 Array | 24 |  |
| GSE182270 | Ulcerative Colitis is characterized by an intense and plasmablast skewed humoral response that associates with treatment resistance and disease complications | Kenigsberg E et al. [16] |  | Illumina HiSeq 2000 (Homo sapiens) | 4 | Single-cell RNA-sequencing |
| GSE150115 | Single-Cell Analyses of Colon and Blood Reveal Distinct Immune Cell Signatures of Ulcerative Colitis and Crohn’s Disease | Mitsialis V et al. [17] | 32428507 | Illumina NextSeq 500 (Homo sapiens) | 5 |  |

**Table S2.** Detailed Mayo scores of enrolled patients at baseline and post-treatment

| **Patients** | **Time Point** | **Stool frequency** | **Rectal bleeding** | **Endoscopic subscore** | **Physician’s global assessment** | **Total Mayo score** |
| --- | --- | --- | --- | --- | --- | --- |
| P1 | Baseline | 3 | 2 | 2 | 2 | 9 |
|  | Post-treatment | 1 | 2 | 1 | 2 | 6 |
| P2 | Baseline | 1 | 2 | 2 | 2 | 7 |
|  | Post-treatment | 2 | 0 | 1 | 1 | 4 |
| P3 | Baseline | 3 | 2 | 3 | 3 | 11 |
|  | Post-treatment | 2 | 3 | 3 | 3 | 11 |
| P4 | Baseline | 3 | 2 | 2 | 2 | 9 |
|  | Post-treatment | 1 | 0 | 0 | 1 | 2 |
| P5 | Baseline | 2 | 3 | 2 | 2 | 9 |
|  | Post-treatment | 1 | 1 | 1 | 1 | 4 |
| P6 | Baseline | 2 | 2 | 3 | 3 | 10 |
|  | Post-treatment | 2 | 2 | 3 | 2 | 9 |
| P7 | Baseline | 2 | 2 | 2 | 3 | 9 |
|  | Post-treatment | 0 | 0 | 1 | 0 | 1 |
| P8 | Baseline | 1 | 3 | 2 | 2 | 8 |
|  | Post-treatment | 1 | 0 | 0 | 1 | 2 |
| P9 | Baseline | 3 | 2 | 2 | 2 | 9 |
|  | Post-treatment | 2 | 1 | 2 | 2 | 7 |
| P10 | Baseline | 2 | 3 | 3 | 2 | 10 |
|  | Post-treatment | 3 | 3 | 3 | 3 | 12 |
| P11 | Baseline | 2 | 2 | 2 | 2 | 8 |
|  | Post-treatment | 0 | 0 | 1 | 1 | 2 |
| P12 | Baseline | 3 | 2 | 2 | 2 | 9 |
|  | Post-treatment | 3 | 3 | 2 | 2 | 10 |

**Table S3.** Included therapeutic pathway genes and their source databases

| **Gene** | **Pathway** | **Source** |
| --- | --- | --- |
| ADAMTS1 | Leukotriene and thrombin | C2 CGP UZONYI |
| APOLD1 | Leukotriene and thrombin | C2 CGP UZONYI |
| ARID5B | Leukotriene and thrombin | C2 CGP UZONYI |
| ATF3 | Leukotriene and thrombin | C2 CGP UZONYI |
| BMP2 | Leukotriene and thrombin | C2 CGP UZONYI |
| CCN1 | Leukotriene and thrombin | C2 CGP UZONYI |
| CITED2 | Leukotriene and thrombin | C2 CGP UZONYI |
| CREM | Leukotriene and thrombin | C2 CGP UZONYI |
| CXCL2 | Leukotriene and thrombin | C2 CGP UZONYI |
| CXCL8 | Leukotriene and thrombin | C2 CGP UZONYI |
| DUSP1 | Leukotriene and thrombin | C2 CGP UZONYI |
| EGR1 | Leukotriene and thrombin | C2 CGP UZONYI |
| EGR3 | Leukotriene and thrombin | C2 CGP UZONYI |
| ELL2 | Leukotriene and thrombin | C2 CGP UZONYI |
| F3 | Leukotriene and thrombin | C2 CGP UZONYI |
| FOSL2 | Leukotriene and thrombin | C2 CGP UZONYI |
| GEM | Leukotriene and thrombin | C2 CGP UZONYI |
| HBEGF | Leukotriene and thrombin | C2 CGP UZONYI |
| HLX | Leukotriene and thrombin | C2 CGP UZONYI |
| KCNJ2 | Leukotriene and thrombin | C2 CGP UZONYI |
| KLF2 | Leukotriene and thrombin | C2 CGP UZONYI |
| KLF4 | Leukotriene and thrombin | C2 CGP UZONYI |
| MAP3K8 | Leukotriene and thrombin | C2 CGP UZONYI |
| MCL1 | Leukotriene and thrombin | C2 CGP UZONYI |
| NDRG1 | Leukotriene and thrombin | C2 CGP UZONYI |
| NEDD9 | Leukotriene and thrombin | C2 CGP UZONYI |
| NR4A1 | Leukotriene and thrombin | C2 CGP UZONYI |
| NR4A2 | Leukotriene and thrombin | C2 CGP UZONYI |
| NR4A3 | Leukotriene and thrombin | C2 CGP UZONYI |
| PDK4 | Leukotriene and thrombin | C2 CGP UZONYI |
| PTGS2 | Leukotriene and thrombin | C2 CGP UZONYI |
| RCAN1 | Leukotriene and thrombin | C2 CGP UZONYI |
| RGS2 | Leukotriene and thrombin | C2 CGP UZONYI |
| SELE | Leukotriene and thrombin | C2 CGP UZONYI |
| SIK1 | Leukotriene and thrombin | C2 CGP UZONYI |
| ZFP36 | Leukotriene and thrombin | C2 CGP UZONYI |
| AKR1B1 | Prostaglandin | WP98 |
| AKR1C1 | Prostaglandin | WP98 |
| AKR1C2 | Prostaglandin | WP98 |
| AKR1C3 | Prostaglandin | WP98 |
| ANXA1 | Prostaglandin | WP98 |
| ANXA2 | Prostaglandin | WP98 |
| ANXA3 | Prostaglandin | WP98 |
| ANXA4 | Prostaglandin | WP98 |
| ANXA5 | Prostaglandin | WP98 |
| ANXA6 | Prostaglandin | WP98 |
| CBR1 | Prostaglandin | WP98 |
| CYP11A1 | Prostaglandin | WP98 |
| EDN1 | Prostaglandin | WP98 |
| EDNRA | Prostaglandin | WP98 |
| EDNRB | Prostaglandin | WP98 |
| HPGD | Prostaglandin | WP98 |
| HPGDS | Prostaglandin | WP98 |
| HSD11B1 | Prostaglandin | WP98 |
| HSD11B2 | Prostaglandin | WP98 |
| MITF | Prostaglandin | WP98 |
| PLA2G4A | Prostaglandin | WP98 |
| PPARG | Prostaglandin | WP98 |
| PPARGC1A | Prostaglandin | WP98 |
| PPARGC1B | Prostaglandin | WP98 |
| PRL | Prostaglandin | WP98 |
| PTGDR | Prostaglandin | WP98 |
| PTGDS | Prostaglandin | WP98 |
| PTGER1 | Prostaglandin | WP98 |
| PTGER2 | Prostaglandin | WP98 |
| PTGER3 | Prostaglandin | WP98 |
| PTGER4 | Prostaglandin | WP98 |
| PTGES | Prostaglandin | WP98 |
| PTGFR | Prostaglandin | WP98 |
| PTGFRN | Prostaglandin | WP98 |
| PTGIR | Prostaglandin | WP98 |
| PTGIS | Prostaglandin | WP98 |
| PTGS1 | Prostaglandin | WP98 |
| PTGS2 | Prostaglandin | WP98 |
| S100A10 | Prostaglandin | WP98 |
| S100A6 | Prostaglandin | WP98 |
| SCGB1A1 | Prostaglandin | WP98 |
| SOX9 | Prostaglandin | WP98 |
| TBXA2R | Prostaglandin | WP98 |
| TBXAS1 | Prostaglandin | WP98 |
| ADAMTS18 | Platelet activation | GO:0010543 |
| ALOX12 | Platelet activation | GO:0010543 |
| APOE | Platelet activation | GO:0010543 |
| BLK | Platelet activation | GO:0010543 |
| C1QTNF1 | Platelet activation | GO:0010543 |
| CD9 | Platelet activation | GO:0010543 |
| CEACAM1 | Platelet activation | GO:0010543 |
| CELA2A | Platelet activation | GO:0010543 |
| DMTN | Platelet activation | GO:0010543 |
| F11R | Platelet activation | GO:0010543 |
| F2 | Platelet activation | GO:0010543 |
| FCER1G | Platelet activation | GO:0010543 |
| HRG | Platelet activation | GO:0010543 |
| LYN | Platelet activation | GO:0010543 |
| NOS3 | Platelet activation | GO:0010543 |
| PDGFA | Platelet activation | GO:0010543 |
| PDGFB | Platelet activation | GO:0010543 |
| PDGFRA | Platelet activation | GO:0010543 |
| PDPN | Platelet activation | GO:0010543 |
| PLA2G4A | Platelet activation | GO:0010543 |
| PLEK | Platelet activation | GO:0010543 |
| PRKCA | Platelet activation | GO:0010543 |
| PRKCD | Platelet activation | GO:0010543 |
| PRKCQ | Platelet activation | GO:0010543 |
| PRKG1 | Platelet activation | GO:0010543 |
| SELP | Platelet activation | GO:0010543 |
| SERPINE2 | Platelet activation | GO:0010543 |
| SH2B3 | Platelet activation | GO:0010543 |
| SYK | Platelet activation | GO:0010543 |
| TEC | Platelet activation | GO:0010543 |
| THBD | Platelet activation | GO:0010543 |
| TLR4 | Platelet activation | GO:0010543 |
| TXK | Platelet activation | GO:0010543 |
| UBASH3B | Platelet activation | GO:0010543 |
| CHPT1 | Platelet activation | GO:0046469 |
| LPCAT2 | Platelet activation | GO:0046470 |
| PAFAH1B1 | Platelet activation | GO:0046471 |
| PLA2G10 | Platelet activation | GO:0046472 |
| PLA2G4C | Platelet activation | GO:0046474 |
| PLA2G6 | Platelet activation | GO:0046475 |
| PLA2G7 | Platelet activation | GO:0046476 |
| ADAM8 | TNF | GO:1903557 |
| AGER | TNF | GO:1903557 |
| APP | TNF | GO:1903557 |
| ARFGEF2 | TNF | GO:1903557 |
| ARHGEF2 | TNF | GO:1903557 |
| AZU1 | TNF | GO:1903557 |
| BCL10 | TNF | GO:1903557 |
| C1QTNF4 | TNF | GO:1903557 |
| CARD9 | TNF | GO:1903557 |
| CCL19 | TNF | GO:1903557 |
| CCL3 | TNF | GO:1903557 |
| CCR2 | TNF | GO:1903557 |
| CD14 | TNF | GO:1903557 |
| CD2 | TNF | GO:1903557 |
| CD36 | TNF | GO:1903557 |
| CD86 | TNF | GO:1903557 |
| CLEC7A | TNF | GO:1903557 |
| CLU | TNF | GO:1903557 |
| CYBA | TNF | GO:1903557 |
| DDT | TNF | GO:1903557 |
| DDX58 | TNF | GO:1903557 |
| DHX9 | TNF | GO:1903557 |
| FADD | TNF | GO:1903557 |
| FRMD8 | TNF | GO:1903557 |
| FZD5 | TNF | GO:1903557 |
| HAVCR2 | TNF | GO:1903557 |
| HDAC2 | TNF | GO:1903557 |
| HLA-E | TNF | GO:1903557 |
| HMGB1 | TNF | GO:1903557 |
| HSPB1 | TNF | GO:1903557 |
| IFIH1 | TNF | GO:1903557 |
| IFNG | TNF | GO:1903557 |
| IFNGR1 | TNF | GO:1903557 |
| IL12B | TNF | GO:1903557 |
| IL17A | TNF | GO:1903557 |
| IL1A | TNF | GO:1903557 |
| IL23A | TNF | GO:1903557 |
| IL33 | TNF | GO:1903557 |
| IL6 | TNF | GO:1903557 |
| ISL1 | TNF | GO:1903557 |
| JAK2 | TNF | GO:1903557 |
| LBP | TNF | GO:1903557 |
| LEP | TNF | GO:1903557 |
| LGALS9 | TNF | GO:1903557 |
| LILRA2 | TNF | GO:1903557 |
| LILRA5 | TNF | GO:1903557 |
| LPL | TNF | GO:1903557 |
| LY96 | TNF | GO:1903557 |
| MAPKAPK2 | TNF | GO:1903557 |
| MAVS | TNF | GO:1903557 |
| MIF | TNF | GO:1903557 |
| MIR144 | TNF | GO:1903557 |
| MIR206 | TNF | GO:1903557 |
| MIR27B | TNF | GO:1903557 |
| MIR657 | TNF | GO:1903557 |
| MMP8 | TNF | GO:1903557 |
| MYD88 | TNF | GO:1903557 |
| NFATC4 | TNF | GO:1903557 |
| NOD1 | TNF | GO:1903557 |
| NOD2 | TNF | GO:1903557 |
| ORM1 | TNF | GO:1903557 |
| ORM2 | TNF | GO:1903557 |
| PF4 | TNF | GO:1903557 |
| PIK3R1 | TNF | GO:1903557 |
| PSEN1 | TNF | GO:1903557 |
| PTAFR | TNF | GO:1903557 |
| PTPN11 | TNF | GO:1903557 |
| PTPRC | TNF | GO:1903557 |
| PTPRJ | TNF | GO:1903557 |
| PYCARD | TNF | GO:1903557 |
| RASGRP1 | TNF | GO:1903557 |
| RIPK1 | TNF | GO:1903557 |
| RIPK2 | TNF | GO:1903557 |
| SASH3 | TNF | GO:1903557 |
| SELENOK | TNF | GO:1903557 |
| SPN | TNF | GO:1903557 |
| STAT3 | TNF | GO:1903557 |
| SYK | TNF | GO:1903557 |
| THBS1 | TNF | GO:1903557 |
| TICAM1 | TNF | GO:1903557 |
| TIRAP | TNF | GO:1903557 |
| TLR1 | TNF | GO:1903557 |
| TLR2 | TNF | GO:1903557 |
| TLR3 | TNF | GO:1903557 |
| TLR4 | TNF | GO:1903557 |
| TLR9 | TNF | GO:1903557 |
| TNFRSF8 | TNF | GO:1903557 |
| TWIST1 | TNF | GO:1903557 |
| TYROBP | TNF | GO:1903557 |
| WNT5A | TNF | GO:1903557 |
| ABL1 | Integrin | GO:0007229 |
| ADAM10 | Integrin | GO:0007229 |
| ADAM11 | Integrin | GO:0007229 |
| ADAM15 | Integrin | GO:0007229 |
| ADAM9 | Integrin | GO:0007229 |
| ADAMTS1 | Integrin | GO:0007229 |
| ADAMTS13 | Integrin | GO:0007229 |
| ANGPTL3 | Integrin | GO:0007229 |
| APOA1 | Integrin | GO:0007229 |
| BCAR1 | Integrin | GO:0007229 |
| BST1 | Integrin | GO:0007229 |
| CCM2 | Integrin | GO:0007229 |
| CCN2 | Integrin | GO:0007229 |
| CD177 | Integrin | GO:0007229 |
| CD40LG | Integrin | GO:0007229 |
| CD47 | Integrin | GO:0007229 |
| CD63 | Integrin | GO:0007229 |
| CDC42 | Integrin | GO:0007229 |
| CDH17 | Integrin | GO:0007229 |
| CEACAM1 | Integrin | GO:0007229 |
| COL16A1 | Integrin | GO:0007229 |
| COL3A1 | Integrin | GO:0007229 |
| CTNNA1 | Integrin | GO:0007229 |
| CUL3 | Integrin | GO:0007229 |
| DMTN | Integrin | GO:0007229 |
| DOCK1 | Integrin | GO:0007229 |
| DST | Integrin | GO:0007229 |
| EMP2 | Integrin | GO:0007229 |
| ERBIN | Integrin | GO:0007229 |
| FERMT1 | Integrin | GO:0007229 |
| FERMT2 | Integrin | GO:0007229 |
| FERMT3 | Integrin | GO:0007229 |
| FGR | Integrin | GO:0007229 |
| FLNA | Integrin | GO:0007229 |
| FN1 | Integrin | GO:0007229 |
| FUT8 | Integrin | GO:0007229 |
| FYB1 | Integrin | GO:0007229 |
| FYB2 | Integrin | GO:0007229 |
| HCK | Integrin | GO:0007229 |
| ILK | Integrin | GO:0007229 |
| ISG15 | Integrin | GO:0007229 |
| ITGA1 | Integrin | GO:0007229 |
| ITGA10 | Integrin | GO:0007229 |
| ITGA11 | Integrin | GO:0007229 |
| ITGA2 | Integrin | GO:0007229 |
| ITGA2B | Integrin | GO:0007229 |
| ITGA3 | Integrin | GO:0007229 |
| ITGA4 | Integrin | GO:0007229 |
| ITGA5 | Integrin | GO:0007229 |
| ITGA6 | Integrin | GO:0007229 |
| ITGA7 | Integrin | GO:0007229 |
| ITGA8 | Integrin | GO:0007229 |
| ITGA9 | Integrin | GO:0007229 |
| ITGAD | Integrin | GO:0007229 |
| ITGAE | Integrin | GO:0007229 |
| ITGAL | Integrin | GO:0007229 |
| ITGAM | Integrin | GO:0007229 |
| ITGAV | Integrin | GO:0007229 |
| ITGAX | Integrin | GO:0007229 |
| ITGB1 | Integrin | GO:0007229 |
| ITGB1BP1 | Integrin | GO:0007229 |
| ITGB2 | Integrin | GO:0007229 |
| ITGB3 | Integrin | GO:0007229 |
| ITGB4 | Integrin | GO:0007229 |
| ITGB5 | Integrin | GO:0007229 |
| ITGB6 | Integrin | GO:0007229 |
| ITGB7 | Integrin | GO:0007229 |
| ITGB8 | Integrin | GO:0007229 |
| ITGBL1 | Integrin | GO:0007229 |
| LAMA3 | Integrin | GO:0007229 |
| LAMA5 | Integrin | GO:0007229 |
| LAT | Integrin | GO:0007229 |
| LIMS1 | Integrin | GO:0007229 |
| LIMS2 | Integrin | GO:0007229 |
| LOXL3 | Integrin | GO:0007229 |
| MADCAM1 | Integrin | GO:0007229 |
| MPIG6B | Integrin | GO:0007229 |
| MYH9 | Integrin | GO:0007229 |
| NEDD9 | Integrin | GO:0007229 |
| NME2 | Integrin | GO:0007229 |
| NRP1 | Integrin | GO:0007229 |
| PHACTR4 | Integrin | GO:0007229 |
| PLEK | Integrin | GO:0007229 |
| PLPP3 | Integrin | GO:0007229 |
| PRAM1 | Integrin | GO:0007229 |
| PRKD1 | Integrin | GO:0007229 |
| PTK2 | Integrin | GO:0007229 |
| PTK2B | Integrin | GO:0007229 |
| PTN | Integrin | GO:0007229 |
| PTPN11 | Integrin | GO:0007229 |
| PTPRA | Integrin | GO:0007229 |
| RCC2 | Integrin | GO:0007229 |
| SEMA7A | Integrin | GO:0007229 |
| SLC2A10 | Integrin | GO:0007229 |
| SRC | Integrin | GO:0007229 |
| SYK | Integrin | GO:0007229 |
| TEC | Integrin | GO:0007229 |
| THY1 | Integrin | GO:0007229 |
| TIMP1 | Integrin | GO:0007229 |
| TLN1 | Integrin | GO:0007229 |
| TSPAN32 | Integrin | GO:0007229 |
| TXK | Integrin | GO:0007229 |
| VAV1 | Integrin | GO:0007229 |
| VAV3 | Integrin | GO:0007229 |
| ZNF304 | Integrin | GO:0007229 |
| ZYX | Integrin | GO:0007229 |
| AGT | Janus Kinase | GO:0042976 |
| CCL5 | Janus Kinase | GO:0042976 |
| CD300A | Janus Kinase | GO:0042976 |
| GH1 | Janus Kinase | GO:0042976 |
| GHR | Janus Kinase | GO:0042976 |
| IL12B | Janus Kinase | GO:0042976 |
| IL23A | Janus Kinase | GO:0042976 |
| IL23R | Janus Kinase | GO:0042976 |
| IL4 | Janus Kinase | GO:0042976 |
| IL6R | Janus Kinase | GO:0042976 |
| JAK2 | Janus Kinase | GO:0042976 |
| PIBF1 | Janus Kinase | GO:0042976 |
| PRLR | Janus Kinase | GO:0042976 |
| PTK2B | Janus Kinase | GO:0042976 |
| SOCS1 | Janus Kinase | GO:0042976 |

**Table S4.** Comparison of clinical traits between 3 clusters

|  | **Cluster1** | **Cluster2** | **Cluster3** | **p.overall** |
| --- | --- | --- | --- | --- |
|  | N=109 | N=62 | N=37 |  |
| GEO session: |  |  |  | 0.496 |
| GSE107499 | 26 (23.9%) | 10 (16.1%) | 11 (29.7%) |  |
| GSE75214 | 37 (33.9%) | 23 (37.1%) | 14 (37.8%) |  |
| GSE87466 | 46 (42.2%) | 29 (46.8%) | 12 (32.4%) |  |
| Gender: |  |  |  | 0.201 |
| Female | 17 (65.4%) | 4 (40.0%) | 4 (36.4%) |  |
| Male | 9 (34.6%) | 6 (60.0%) | 7 (63.6%) |  |
| age | 39.2 (16.4) | 39.6 (13.7) | 33.5 (13.2) | 0.25 |
| Location: |  |  |  | 0.127 |
| Ascending | 0 (0.00%) | 0 (0.00%) | 2 (18.2%) |  |
| Descending | 6 (23.1%) | 4 (40.0%) | 2 (18.2%) |  |
| Sigmoid | 13 (50.0%) | 5 (50.0%) | 7 (63.6%) |  |
| Transverse | 7 (26.9%) | 1 (10.0%) | 0 (0.00%) |  |
| Severity: |  |  |  | 0.262 |
| Extensive | 14 (30.4%) | 7 (24.1%) | 6 (50.0%) |  |
| Limited | 32 (69.6%) | 22 (75.9%) | 6 (50.0%) |  |

**Table S5.** GO enrichment for each WGCNA module

| **Cluster** | **ID** | **Description** | **GeneRatio** | **pvalue** | **FDR** | **qvalue** |
| --- | --- | --- | --- | --- | --- | --- |
| black | GO:0030098 | lymphocyte differentiation | 21/304 | 0.000 | 0.002 | 0.002 |
| black | GO:0001776 | leukocyte homeostasis | 10/304 | 0.000 | 0.002 | 0.002 |
| black | GO:0042116 | macrophage activation | 10/304 | 0.000 | 0.004 | 0.004 |
| black | GO:1900242 | regulation of synaptic vesicle endocytosis | 5/304 | 0.000 | 0.004 | 0.004 |
| black | GO:1903039 | positive regulation of leukocyte cell-cell adhesion | 15/304 | 0.000 | 0.004 | 0.004 |
| black | GO:0042113 | B cell activation | 18/304 | 0.000 | 0.004 | 0.004 |
| black | GO:0030217 | T cell differentiation | 14/304 | 0.000 | 0.014 | 0.013 |
| black | GO:0043087 | regulation of GTPase activity | 20/304 | 0.000 | 0.021 | 0.019 |
| black | GO:0042110 | T cell activation | 19/304 | 0.000 | 0.043 | 0.039 |
| blue | GO:0007015 | actin filament organization | 33/569 | 0.000 | 0.005 | 0.005 |
| blue | GO:0044282 | small molecule catabolic process | 32/569 | 0.000 | 0.018 | 0.016 |
| blue | GO:0019318 | hexose metabolic process | 21/569 | 0.000 | 0.045 | 0.041 |
| blue | GO:0032970 | regulation of actin filament-based process | 28/569 | 0.000 | 0.045 | 0.041 |
| blue | GO:0007034 | vacuolar transport | 15/569 | 0.000 | 0.045 | 0.041 |
| brown | GO:0009259 | ribonucleotide metabolic process | 27/431 | 0.000 | 0.003 | 0.002 |
| brown | GO:0019693 | ribose phosphate metabolic process | 27/431 | 0.000 | 0.003 | 0.002 |
| brown | GO:0033865 | nucleoside bisphosphate metabolic process | 14/431 | 0.000 | 0.003 | 0.002 |
| brown | GO:0033875 | ribonucleoside bisphosphate metabolic process | 14/431 | 0.000 | 0.003 | 0.002 |
| brown | GO:0034032 | purine nucleoside bisphosphate metabolic process | 14/431 | 0.000 | 0.003 | 0.002 |
| green | GO:0030098 | lymphocyte differentiation | 37/324 | 0.000 | 0.000 | 0.000 |
| green | GO:0042110 | T cell activation | 39/324 | 0.000 | 0.000 | 0.000 |
| green | GO:0042113 | B cell activation | 31/324 | 0.000 | 0.000 | 0.000 |
| green | GO:0030217 | T cell differentiation | 27/324 | 0.000 | 0.000 | 0.000 |
| green | GO:0002460 | adaptive immune response based on somatic recombination of immune receptors built from immunoglobulin superfamily domains | 30/324 | 0.000 | 0.000 | 0.000 |
| green | GO:1903039 | positive regulation of leukocyte cell-cell adhesion | 21/324 | 0.000 | 0.000 | 0.000 |
| green | GO:0001776 | leukocyte homeostasis | 10/324 | 0.000 | 0.000 | 0.000 |
| green | GO:0043087 | regulation of GTPase activity | 20/324 | 0.000 | 0.006 | 0.005 |
| green | GO:0001819 | positive regulation of cytokine production | 19/324 | 0.000 | 0.007 | 0.006 |
| green | GO:0043312 | neutrophil degranulation | 18/324 | 0.002 | 0.031 | 0.027 |
| green | GO:0002283 | neutrophil activation involved in immune response | 18/324 | 0.002 | 0.032 | 0.028 |
| greenyellow | GO:0030198 | extracellular matrix organization | 32/193 | 0.000 | 0.000 | 0.000 |
| greenyellow | GO:0043062 | extracellular structure organization | 32/193 | 0.000 | 0.000 | 0.000 |
| greenyellow | GO:0030199 | collagen fibril organization | 8/193 | 0.000 | 0.000 | 0.000 |
| greenyellow | GO:1901342 | regulation of vasculature development | 17/193 | 0.000 | 0.002 | 0.002 |
| greenyellow | GO:0045765 | regulation of angiogenesis | 16/193 | 0.000 | 0.002 | 0.002 |
| greenyellow | GO:0031589 | cell-substrate adhesion | 14/193 | 0.000 | 0.004 | 0.004 |
| greenyellow | GO:0001667 | ameboidal-type cell migration | 14/193 | 0.000 | 0.042 | 0.038 |
| grey | GO:0001655 | urogenital system development | 50/1115 | 0.000 | 0.000 | 0.000 |
| grey | GO:0072001 | renal system development | 44/1115 | 0.000 | 0.000 | 0.000 |
| grey | GO:0001822 | kidney development | 42/1115 | 0.000 | 0.000 | 0.000 |
| grey | GO:0072073 | kidney epithelium development | 26/1115 | 0.000 | 0.000 | 0.000 |
| grey | GO:0072006 | nephron development | 26/1115 | 0.000 | 0.000 | 0.000 |
| grey | GO:0055088 | lipid homeostasis | 21/1115 | 0.001 | 0.035 | 0.031 |
| magenta | GO:0061952 | midbody abscission | 6/244 | 0.000 | 0.000 | 0.000 |
| magenta | GO:1902410 | mitotic cytokinetic process | 6/244 | 0.000 | 0.001 | 0.001 |
| magenta | GO:0046755 | viral budding | 6/244 | 0.000 | 0.001 | 0.001 |
| magenta | GO:0007032 | endosome organization | 9/244 | 0.000 | 0.001 | 0.001 |
| magenta | GO:1901673 | regulation of mitotic spindle assembly | 5/244 | 0.000 | 0.001 | 0.001 |
| pink | GO:0002283 | neutrophil activation involved in immune response | 53/245 | 0.000 | 0.000 | 0.000 |
| pink | GO:0043312 | neutrophil degranulation | 52/245 | 0.000 | 0.000 | 0.000 |
| pink | GO:0002237 | response to molecule of bacterial origin | 40/245 | 0.000 | 0.000 | 0.000 |
| pink | GO:0032496 | response to lipopolysaccharide | 39/245 | 0.000 | 0.000 | 0.000 |
| pink | GO:0001819 | positive regulation of cytokine production | 43/245 | 0.000 | 0.000 | 0.000 |
| pink | GO:0042116 | macrophage activation | 16/245 | 0.000 | 0.000 | 0.000 |
| pink | GO:0051092 | positive regulation of NF-kappaB transcription factor activity | 18/245 | 0.000 | 0.000 | 0.000 |
| pink | GO:0042110 | T cell activation | 29/245 | 0.000 | 0.000 | 0.000 |
| pink | GO:1903039 | positive regulation of leukocyte cell-cell adhesion | 19/245 | 0.000 | 0.000 | 0.000 |
| pink | GO:0030098 | lymphocyte differentiation | 19/245 | 0.000 | 0.000 | 0.000 |
| pink | GO:0001776 | leukocyte homeostasis | 9/245 | 0.000 | 0.000 | 0.000 |
| pink | GO:0030198 | extracellular matrix organization | 18/245 | 0.000 | 0.000 | 0.000 |
| pink | GO:0043062 | extracellular structure organization | 18/245 | 0.000 | 0.000 | 0.000 |
| pink | GO:0030217 | T cell differentiation | 14/245 | 0.000 | 0.000 | 0.000 |
| pink | GO:0002460 | adaptive immune response based on somatic recombination of immune receptors built from immunoglobulin superfamily domains | 16/245 | 0.000 | 0.000 | 0.000 |
| pink | GO:0001890 | placenta development | 9/245 | 0.000 | 0.002 | 0.002 |
| pink | GO:1901342 | regulation of vasculature development | 16/245 | 0.000 | 0.003 | 0.002 |
| pink | GO:0045765 | regulation of angiogenesis | 15/245 | 0.000 | 0.003 | 0.002 |
| pink | GO:0042113 | B cell activation | 12/245 | 0.001 | 0.010 | 0.007 |
| red | GO:0006066 | alcohol metabolic process | 20/307 | 0.000 | 0.019 | 0.018 |
| tan | GO:0045333 | cellular respiration | 41/166 | 0.000 | 0.000 | 0.000 |
| tan | GO:0022900 | electron transport chain | 38/166 | 0.000 | 0.000 | 0.000 |
| tan | GO:0022904 | respiratory electron transport chain | 33/166 | 0.000 | 0.000 | 0.000 |
| tan | GO:0042775 | mitochondrial ATP synthesis coupled electron transport | 31/166 | 0.000 | 0.000 | 0.000 |
| tan | GO:0042773 | ATP synthesis coupled electron transport | 31/166 | 0.000 | 0.000 | 0.000 |
| tan | GO:0016054 | organic acid catabolic process | 10/166 | 0.000 | 0.008 | 0.008 |
| tan | GO:0046395 | carboxylic acid catabolic process | 10/166 | 0.000 | 0.008 | 0.008 |
| tan | GO:0033865 | nucleoside bisphosphate metabolic process | 7/166 | 0.000 | 0.008 | 0.008 |
| tan | GO:0033875 | ribonucleoside bisphosphate metabolic process | 7/166 | 0.000 | 0.008 | 0.008 |
| tan | GO:0034032 | purine nucleoside bisphosphate metabolic process | 7/166 | 0.000 | 0.008 | 0.008 |
| tan | GO:0009259 | ribonucleotide metabolic process | 11/166 | 0.001 | 0.031 | 0.029 |
| tan | GO:0019693 | ribose phosphate metabolic process | 11/166 | 0.001 | 0.036 | 0.033 |
| tan | GO:0044282 | small molecule catabolic process | 11/166 | 0.002 | 0.049 | 0.046 |
| turquoise | GO:0072329 | monocarboxylic acid catabolic process | 24/579 | 0.000 | 0.000 | 0.000 |
| turquoise | GO:0016054 | organic acid catabolic process | 34/579 | 0.000 | 0.000 | 0.000 |
| turquoise | GO:0046395 | carboxylic acid catabolic process | 34/579 | 0.000 | 0.000 | 0.000 |
| turquoise | GO:0044282 | small molecule catabolic process | 43/579 | 0.000 | 0.000 | 0.000 |
| turquoise | GO:0009062 | fatty acid catabolic process | 20/579 | 0.000 | 0.000 | 0.000 |
| turquoise | GO:0006066 | alcohol metabolic process | 31/579 | 0.000 | 0.000 | 0.000 |
| turquoise | GO:0022904 | respiratory electron transport chain | 15/579 | 0.000 | 0.001 | 0.001 |
| turquoise | GO:0045333 | cellular respiration | 19/579 | 0.000 | 0.001 | 0.001 |
| turquoise | GO:0033865 | nucleoside bisphosphate metabolic process | 16/579 | 0.000 | 0.001 | 0.001 |
| turquoise | GO:0033875 | ribonucleoside bisphosphate metabolic process | 16/579 | 0.000 | 0.001 | 0.001 |
| turquoise | GO:0034032 | purine nucleoside bisphosphate metabolic process | 16/579 | 0.000 | 0.001 | 0.001 |
| turquoise | GO:0042775 | mitochondrial ATP synthesis coupled electron transport | 13/579 | 0.000 | 0.001 | 0.001 |
| turquoise | GO:0042773 | ATP synthesis coupled electron transport | 13/579 | 0.000 | 0.001 | 0.001 |
| turquoise | GO:0022900 | electron transport chain | 16/579 | 0.000 | 0.012 | 0.012 |
| turquoise | GO:0019318 | hexose metabolic process | 19/579 | 0.000 | 0.018 | 0.017 |
| turquoise | GO:0006821 | chloride transport | 11/579 | 0.001 | 0.029 | 0.027 |
| yellow | GO:0030198 | extracellular matrix organization | 68/366 | 0.000 | 0.000 | 0.000 |
| yellow | GO:0043062 | extracellular structure organization | 68/366 | 0.000 | 0.000 | 0.000 |
| yellow | GO:0001667 | ameboidal-type cell migration | 46/366 | 0.000 | 0.000 | 0.000 |
| yellow | GO:0031589 | cell-substrate adhesion | 40/366 | 0.000 | 0.000 | 0.000 |
| yellow | GO:0090130 | tissue migration | 40/366 | 0.000 | 0.000 | 0.000 |
| yellow | GO:0030199 | collagen fibril organization | 15/366 | 0.000 | 0.000 | 0.000 |
| yellow | GO:0072001 | renal system development | 29/366 | 0.000 | 0.000 | 0.000 |
| yellow | GO:0001655 | urogenital system development | 30/366 | 0.000 | 0.000 | 0.000 |
| yellow | GO:0045765 | regulation of angiogenesis | 33/366 | 0.000 | 0.000 | 0.000 |
| yellow | GO:1901342 | regulation of vasculature development | 34/366 | 0.000 | 0.000 | 0.000 |
| yellow | GO:0001822 | kidney development | 26/366 | 0.000 | 0.000 | 0.000 |
| yellow | GO:0072006 | nephron development | 17/366 | 0.000 | 0.000 | 0.000 |
| yellow | GO:0032970 | regulation of actin filament-based process | 27/366 | 0.000 | 0.000 | 0.000 |
| yellow | GO:0032496 | response to lipopolysaccharide | 19/366 | 0.000 | 0.000 | 0.000 |
| yellow | GO:0002237 | response to molecule of bacterial origin | 19/366 | 0.000 | 0.001 | 0.001 |
| yellow | GO:0007015 | actin filament organization | 21/366 | 0.000 | 0.001 | 0.001 |
| yellow | GO:0001890 | placenta development | 11/366 | 0.000 | 0.002 | 0.002 |
| yellow | GO:0072073 | kidney epithelium development | 10/366 | 0.000 | 0.004 | 0.003 |
| yellow | GO:0043087 | regulation of GTPase activity | 18/366 | 0.006 | 0.032 | 0.023 |

**Table S6.** Marker genes of each CrossICC subtype

| **Subtype** | **Marker** |
| --- | --- |
| K1 | GBP5 |
| K1 | OSM |
| K1 | SOCS3 |
| K1 | TNF |
| K1 | IDO1 |
| K1 | P2RY13 |
| K1 | TLR1 |
| K1 | PLEK |
| K1 | CLEC4D |
| K1 | CXCL10 |
| K1 | IL1A |
| K1 | IL1B |
| K1 | MX1 |
| K1 | HCK |
| K1 | BCL2A1 |
| K1 | NR1I2 |
| K1 | CXCL9 |
| K1 | LILRA1 |
| K1 | CXCL11 |
| K1 | KCNJ15 |
| K1 | CSF3R |
| K1 | NR4A3 |
| K1 | ICAM1 |
| K1 | LCP2 |
| K1 | MX2 |
| K1 | CLEC7A |
| K1 | MNDA |
| K1 | LILRB2 |
| K1 | IFI44L |
| K1 | FPR2 |
| K1 | PDE4B |
| K1 | G0S2 |
| K1 | CCR1 |
| K1 | PROK2 |
| K1 | SELPLG |
| K1 | CREB5 |
| K1 | LDHD |
| K1 | FPR1 |
| K1 | CXCR1 |
| K1 | TREM1 |
| K1 | CXCR2 |
| K1 | LILRB1 |
| K1 | BCL6 |
| K1 | PKIB |
| K1 | GPR84 |
| K1 | TAGAP |
| K1 | S100A9 |
| K1 | S100A8 |
| K1 | SNX10 |
| K1 | MEP1B |
| K1 | AQP9 |
| K1 | IL1RN |
| K1 | TMEM71 |
| K1 | TLR8 |
| K1 | CLEC4E |
| K1 | TNFAIP6 |
| K1 | VNN2 |
| K1 | PTGS2 |
| K1 | CLEC4A |
| K1 | LILRA3 |
| K1 | S100A12 |
| K1 | DSE |
| K1 | KYNU |
| K1 | CYP2C18 |
| K1 | GPR65 |
| K1 | TNFRSF11B |
| K2 | ITGAM |
| K2 | CR1 |
| K2 | SELL |
| K2 | IL24 |
| K2 | C1orf162 |
| K2 | GFPT2 |
| K2 | FMNL3 |
| K2 | PTGFR |
| K2 | SYT11 |
| K2 | ITGB2 |
| K2 | MMP9 |
| K2 | F5 |
| K2 | PRRX1 |
| K2 | LEF1 |
| K2 | CLU |
| K2 | S1PR1 |
| K2 | AFF3 |
| K2 | TRPS1 |
| K2 | GLI3 |
| K2 | ITPR1 |
| K2 | ITGAX |
| K2 | CCR7 |
| K2 | BCAT1 |
| K2 | ELOVL5 |
| K2 | TNFSF13B |
| K2 | DOCK11 |
| K2 | SNX10 |
| K2 | FADS1 |
| K2 | JAK3 |
| K2 | MX2 |
| K2 | TNFRSF9 |
| K2 | BCL6 |
| K2 | TGFB3 |
| K2 | CHI3L2 |
| K2 | DPYSL3 |
| K2 | PRKCB |
| K2 | FYN |
| K2 | TRIB2 |
| K2 | LILRB4 |
| K2 | KLF12 |
| K2 | ITK |
| K2 | SLA |
| K2 | CEP170 |
| K2 | CD48 |
| K2 | RASSF2 |
| K2 | HLA-DOB |
| K2 | CLEC2D |
| K2 | LCP2 |
| K2 | CD53 |
| K2 | CD3D |
| K2 | TMEM119 |
| K2 | LSAMP |
| K2 | KLHL5 |
| K2 | ANXA6 |
| K2 | CD28 |
| K2 | CTSK |
| K2 | TSC22D3 |
| K2 | FAM126A |
| K2 | TMEM163 |
| K2 | TMEM154 |
| K2 | ADAM12 |
| K2 | ARHGAP9 |
| K2 | ATM |
| K2 | IKZF1 |
| K2 | SDK1 |
| K2 | LAMP3 |
| K2 | LRRK2 |
| K2 | TSPYL5 |
| K2 | TRAT1 |
| K2 | PTPRC |
| K2 | ALPK2 |
| K2 | SPOCK2 |
| K2 | EVI2A |
| K2 | EBF1 |
| K2 | IL7R |
| K2 | BCL2A1 |
| K2 | RFTN1 |
| K2 | CCL19 |
| K2 | SERPINB9 |
| K2 | SELPLG |
| K2 | STAP1 |
| K2 | CD3E |
| K2 | MYO5A |
| K2 | CCL21 |
| K2 | CD84 |
| K2 | FGF2 |
| K2 | IL33 |
| K2 | VGLL3 |
| K2 | CD2 |
| K2 | AIM2 |
| K2 | GPR65 |
| K2 | HCLS1 |
| K2 | FCRLA |
| K2 | FLRT2 |
| K2 | APBB1IP |
| K2 | SPP1 |
| K2 | WDFY4 |
| K2 | GLIS3 |
| K2 | SERPINF1 |
| K2 | SLAMF1 |
| K2 | LAPTM5 |
| K2 | PLEK |
| K2 | DOCK2 |
| K2 | GMFG |
| K2 | FGR |
| K2 | SEMA7A |
| K2 | FAM171B |
| K2 | ZNF382 |
| K2 | GPR183 |
| K2 | CD80 |
| K2 | SLC16A6 |
| K2 | PDPN |
| K2 | IL2RA |
| K2 | ITGA4 |
| K2 | DSE |
| K2 | SLAMF6 |
| K2 | CHN1 |
| K2 | BNC2 |
| K2 | LYVE1 |
| K2 | LTBP2 |
| K2 | ITGAL |
| K2 | RASGRP1 |
| K2 | NCKAP1L |
| K2 | ISLR |
| K2 | BANK1 |
| K2 | VNN2 |
| K2 | C3 |
| K2 | ST3GAL1 |
| K2 | SLAIN1 |
| K2 | SELP |
| K2 | ST8SIA4 |
| K2 | TIMP3 |
| K2 | CCDC80 |
| K2 | ITGA5 |
| K2 | PLA1A |
| K2 | CHRDL2 |
| K2 | PRKCH |
| K2 | TSPAN11 |
| K2 | CDK14 |
| K2 | TLR2 |
| K2 | SLC16A4 |
| K2 | DOCK8 |
| K2 | RASGRP3 |
| K2 | ANGPTL2 |
| K2 | PARP15 |
| K2 | ELMO1 |
| K2 | OLFML2B |
| K2 | ICAM1 |
| K2 | THY1 |
| K2 | RERG |
| K2 | ZEB1 |
| K2 | ERG |
| K2 | TNS1 |
| K2 | ANTXR1 |
| K2 | CDH11 |
| K2 | WWTR1 |
| K2 | PCOLCE |
| K2 | CYYR1 |
| K2 | NNMT |
| K2 | BGN |
| K2 | CDH5 |
| K2 | CRISPLD2 |
| K2 | RHOJ |
| K2 | SFRP2 |
| K2 | CALCRL |
| K2 | CAV1 |
| K2 | PDGFRB |
| K2 | MOXD1 |
| K2 | FIBIN |
| K2 | FCN3 |
| K2 | FKBP10 |
| K2 | GJA5 |
| K2 | CCDC3 |
| K2 | TDO2 |
| K2 | ZNF521 |
| K2 | CD93 |
| K2 | EDNRA |
| K2 | ARHGAP29 |
| K2 | EGFL6 |
| K2 | TBC1D9 |
| K2 | GREM1 |
| K2 | RSPO3 |
| K2 | PLTP |
| K2 | EHD3 |
| K2 | CLEC2B |
| K2 | COL15A1 |
| K2 | PRICKLE2 |
| K2 | CHST15 |
| K2 | SPON2 |
| K2 | MEIS1 |
| K2 | FAM20C |
| K2 | FNDC1 |
| K2 | DPT |
| K2 | PAPPA |
| K2 | ABI3BP |
| K2 | COL5A2 |
| K2 | LOX |
| K2 | PXDN |
| K2 | LOXL2 |
| K2 | IL10RA |
| K2 | GEM |
| K2 | FGF7 |
| K2 | FAM20A |
| K2 | CCDC69 |
| K2 | VCAN |
| K2 | HGF |
| K2 | NRCAM |
| K2 | GZMK |
| K2 | SRPX2 |
| K2 | SEMA4A |
| K2 | PTP4A3 |
| K2 | PIK3R5 |
| K2 | PREX1 |
| K2 | LY96 |
| K2 | CSGALNACT1 |
| K2 | CCDC88A |
| K2 | IRF4 |
| K2 | FAP |
| K2 | GLCCI1 |
| K2 | THBS2 |
| K2 | FCGR2B |
| K2 | PPP1R16B |
| K2 | CCR1 |
| K2 | MASP1 |
| K2 | CXCL6 |
| K2 | CHI3L1 |
| K2 | TFEC |
| K2 | COL12A1 |
| K2 | SH2D1A |
| K3 | AKAP5 |
| K3 | TBX10 |
| K3 | PRLR |
| K3 | UGT2A3 |
| K3 | HEPACAM2 |
| K3 | THRB |
| K3 | NR5A2 |
| K3 | PCK1 |
| K3 | LRIG3 |
| K3 | WFDC2 |
| K3 | LRRC31 |
| K3 | GUCY2C |
| K3 | MOGAT2 |
| K3 | SAMD13 |
| K3 | DNAJC12 |
| K3 | B3GNT6 |
| K3 | ISX |
| K3 | ANXA13 |
| K3 | TTLL6 |
| K3 | RGMB |
| K3 | SLC4A4 |
| K3 | SATB2 |
| K3 | DDC |
| K3 | SCGB2A1 |
| K3 | PADI2 |
| K3 | EXPH5 |
| K3 | CAPN13 |
| K3 | CHP2 |
| K3 | SEMA4G |
| K3 | UBXN10 |
| K3 | NEDD4L |
| K3 | ST6GALNAC6 |
| K3 | PLCE1 |
| K3 | SLC9A2 |
| K3 | PTGDR |
| K3 | RETNLB |
| K3 | HNF4G |
| K3 | RAVER2 |
| K3 | CAPN9 |
| K3 | IGSF3 |
| K3 | A1CF |
| K3 | VIPR1 |
| K3 | CDHR1 |
| K3 | POF1B |
| K3 | PPARGC1A |
| K3 | B3GNT7 |
| K3 | COBL |
| K3 | RAP1GAP |
| K3 | SLC39A5 |
| K3 | IHH |
| K3 | GSN |
| K3 | LEFTY1 |
| K3 | CYP2J2 |
| K3 | ATP2C2 |
| K3 | CYP4F12 |
| K3 | PCSK6 |
| K3 | CDX2 |
| K3 | ENTPD5 |
| K3 | SOSTDC1 |
| K3 | ACSM3 |
| K3 | FRMD3 |
| K3 | CDKN2B |
| K3 | HMGCS2 |
| K3 | CWH43 |
| K3 | MYO1A |
| K3 | SYTL5 |
| K3 | CKB |
| K3 | DHRS11 |
| K3 | ADH6 |
| K3 | SLC26A2 |
| K3 | ACSF2 |
| K3 | STYK1 |
| K3 | TINAG |
| K3 | FAM160A1 |
| K3 | TMEM171 |
| K3 | STAP2 |
| K3 | EFNA1 |
| K3 | SLC17A4 |
| K3 | HSD17B2 |
| K3 | CA1 |
| K3 | GUCA2A |
| K3 | MEP1A |
| K3 | HSD11B2 |
| K3 | ABCG2 |
| K3 | GLDN |
| K3 | ACVR1C |
| K3 | SULT1B1 |
| K3 | BEST2 |
| K3 | PPARG |
| K3 | PLCB4 |
| K3 | FAM3D |
| K3 | CYP2B6 |
| K3 | VIL1 |
| K3 | RPS6KA6 |
| K3 | LOX |
| K3 | TRPM6 |
| K3 | FABP2 |
| K3 | PDZD3 |
| K3 | PRR5L |
| K3 | FMO5 |
| K3 | MS4A12 |
| K3 | CD177 |
| K3 | GLIS3 |
| K3 | PLEKHG6 |
| K3 | GGT6 |
| K3 | HRCT1 |
| K3 | ENPP3 |
| K3 | ARSD |
| K3 | EDN3 |
| K3 | RASD2 |
| K3 | LRRC19 |
| K3 | SELENBP1 |
| K3 | USP2 |
| K3 | PBLD |
| K3 | STX19 |
| K3 | LDHD |
| K3 | SGK2 |
| K3 | CA4 |
| K3 | CA7 |
| K3 | PIGZ |
| K3 | LRP4 |
| K3 | GUCA2B |
| K3 | CDHR5 |
| K3 | PHLPP2 |
| K3 | PKIB |
| K3 | BEST4 |
| K3 | FMO4 |
| K3 | C10orf99 |
| K3 | PRSS8 |
| K3 | MAP2K6 |
| K3 | HHLA2 |
| K3 | SLITRK6 |
| K3 | SERTAD4 |
| K3 | SCNN1B |
| K3 | TMEM37 |
| K3 | LRRC66 |
| K3 | PLEKHA7 |
| K3 | MYO7B |
| K3 | MUC20 |
| K3 | TUBAL3 |
| K3 | CGN |
| K3 | PTPRR |
| K3 | SLC30A10 |
| K3 | MEP1B |
| K3 | SLC3A1 |
| K3 | TLR3 |
| K3 | HPGD |
| K3 | EGF |
| K3 | MOGAT3 |
| K3 | BTNL3 |
| K3 | PDE9A |
| K3 | TM6SF2 |
| K3 | DQX1 |
| K3 | NR1I2 |
| K3 | CYP2C18 |
| K3 | GDPD2 |
| K3 | IL22RA1 |
| K3 | ALDH1L1 |
| K3 | TRIM31 |
| K3 | SLC15A1 |
| K4 | ORAI2 |
| K4 | CCDC88A |
| K4 | CCDC69 |
| K4 | KLHL6 |
| K4 | FER1L4 |
| K4 | BGN |
| K4 | FMO5 |
| K4 | VCAN |
| K4 | SLC15A1 |
| K4 | LTF |
| K4 | FKBP10 |
| K4 | ICAM2 |
| K4 | IRF4 |
| K4 | SLITRK6 |
| K4 | SLC7A5 |
| K4 | CCDC3 |
| K4 | SERPINF1 |
| K4 | CA4 |
| K4 | THY1 |
| K4 | FADS1 |
| K4 | PDZD3 |
| K4 | PDE9A |
| K4 | TRIM31 |
| K4 | SEMA4A |
| K4 | SPON2 |
| K4 | CXCL13 |
| K4 | PCOLCE |
| K4 | KCNA3 |
| K4 | MAP4K1 |
| K4 | CD72 |
| K4 | RFTN1 |
| K4 | BTK |
| K4 | WDFY4 |
| K4 | CD79B |
| K4 | TCL1A |
| K4 | CD22 |
| K4 | CD19 |
| K4 | ALPK2 |
| K4 | CLU |
| K4 | IL21R |
| K4 | CD180 |
| K4 | CORO1A |
| K4 | NCKAP1L |
| K4 | SP140 |
| K4 | IKZF1 |
| K4 | P2RY8 |
| K4 | SASH3 |
| K4 | ARHGAP25 |
| K4 | JAK3 |
| K4 | HCLS1 |
| K4 | TRAF1 |
| K4 | PTPN6 |

**Table S7.** The CrossICC subtypes of each sample

| **Sample** | **Cluster** |
| --- | --- |
| GSM2869335 | 1 |
| GSM2869336 | 2 |
| GSM2869337 | 3 |
| GSM2869338 | 2 |
| GSM2869339 | 1 |
| GSM2869340 | 4 |
| GSM2869341 | 3 |
| GSM2869342 | 2 |
| GSM2869343 | 2 |
| GSM2869344 | 3 |
| GSM2869345 | 3 |
| GSM2869346 | 1 |
| GSM2869347 | 3 |
| GSM2869349 | 2 |
| GSM2869351 | 3 |
| GSM2869352 | 2 |
| GSM2869353 | 2 |
| GSM2869354 | 2 |
| GSM2869355 | 3 |
| GSM2869356 | 4 |
| GSM2869357 | 2 |
| GSM2869358 | 3 |
| GSM2869362 | 3 |
| GSM2869363 | 3 |
| GSM2869364 | 1 |
| GSM2869365 | 4 |
| GSM2869367 | 2 |
| GSM2869368 | 1 |
| GSM2869391 | 2 |
| GSM2869392 | 3 |
| GSM2869393 | 2 |
| GSM2869394 | 3 |
| GSM2869397 | 2 |
| GSM2869401 | 2 |
| GSM2869404 | 3 |
| GSM2869405 | 3 |
| GSM2869406 | 2 |
| GSM2869408 | 3 |
| GSM2869411 | 2 |
| GSM2869414 | 3 |
| GSM2869419 | 2 |
| GSM2869421 | 2 |
| GSM2869422 | 2 |
| GSM2869424 | 2 |
| GSM2869425 | 2 |
| GSM2869427 | 3 |
| GSM2869430 | 2 |
| GSM1426090 | 3 |
| GSM1426091 | 3 |
| GSM1426092 | 3 |
| GSM1426093 | 3 |
| GSM1426094 | 2 |
| GSM1426095 | 2 |
| GSM1426096 | 1 |
| GSM1426097 | 5 |
| GSM1426098 | 1 |
| GSM1426099 | 1 |
| GSM1426100 | 2 |
| GSM1426101 | 2 |
| GSM1426102 | 3 |
| GSM1426103 | 5 |
| GSM1426104 | 1 |
| GSM1426105 | 2 |
| GSM1426106 | 3 |
| GSM1426107 | 5 |
| GSM1426108 | 1 |
| GSM1426109 | 3 |
| GSM1426110 | 3 |
| GSM1426111 | 3 |
| GSM1426112 | 3 |
| GSM1426113 | 3 |
| GSM1426114 | 2 |
| GSM1426115 | 2 |
| GSM1426116 | 2 |
| GSM1426117 | 3 |
| GSM1426118 | 2 |
| GSM1426119 | 2 |
| GSM1426120 | 3 |
| GSM1426121 | 2 |
| GSM1426122 | 3 |
| GSM1426123 | 3 |
| GSM1426124 | 2 |
| GSM1426125 | 3 |
| GSM1426126 | 2 |
| GSM1426127 | 3 |
| GSM1426128 | 2 |
| GSM1426129 | 2 |
| GSM1426130 | 3 |
| GSM1426131 | 2 |
| GSM1426132 | 2 |
| GSM1426133 | 3 |
| GSM1426134 | 1 |
| GSM1426135 | 3 |
| GSM1426136 | 3 |
| GSM1426137 | 3 |
| GSM1426138 | 2 |
| GSM1426139 | 2 |
| GSM1426140 | 2 |
| GSM1426141 | 3 |
| GSM1426142 | 2 |
| GSM1426143 | 3 |
| GSM1426144 | 3 |
| GSM1426145 | 2 |
| GSM1426146 | 2 |
| GSM1426147 | 3 |
| GSM1426148 | 3 |
| GSM1426149 | 3 |
| GSM1426150 | 2 |
| GSM1426151 | 3 |
| GSM1426152 | 2 |
| GSM1426153 | 2 |
| GSM1426154 | 3 |
| GSM1426155 | 3 |
| GSM1426156 | 3 |
| GSM1426157 | 3 |
| GSM1426158 | 2 |
| GSM1426159 | 2 |
| GSM1426160 | 2 |
| GSM1426161 | 3 |
| GSM1426162 | 2 |
| GSM1426163 | 3 |
| GSM2332119 | 5 |
| GSM2332120 | 2 |
| GSM2332121 | 3 |
| GSM2332122 | 4 |
| GSM2332123 | 4 |
| GSM2332124 | 3 |
| GSM2332125 | 3 |
| GSM2332126 | 3 |
| GSM2332127 | 3 |
| GSM2332128 | 2 |
| GSM2332129 | 3 |
| GSM2332130 | 2 |
| GSM2332131 | 3 |
| GSM2332132 | 3 |
| GSM2332133 | 2 |
| GSM2332134 | 3 |
| GSM2332135 | 2 |
| GSM2332136 | 3 |
| GSM2332137 | 2 |
| GSM2332138 | 3 |
| GSM2332139 | 1 |
| GSM2332140 | 5 |
| GSM2332141 | 5 |
| GSM2332142 | 2 |
| GSM2332143 | 4 |
| GSM2332144 | 2 |
| GSM2332145 | 2 |
| GSM2332146 | 3 |
| GSM2332147 | 1 |
| GSM2332148 | 2 |
| GSM2332149 | 3 |
| GSM2332150 | 3 |
| GSM2332151 | 3 |
| GSM2332152 | 2 |
| GSM2332153 | 3 |
| GSM2332154 | 4 |
| GSM2332155 | 4 |
| GSM2332156 | 4 |
| GSM2332157 | 2 |
| GSM2332158 | 2 |
| GSM2332159 | 2 |
| GSM2332160 | 4 |
| GSM2332161 | 2 |
| GSM2332162 | 2 |
| GSM2332163 | 2 |
| GSM2332164 | 2 |
| GSM2332165 | 2 |
| GSM2332166 | 3 |
| GSM2332167 | 5 |
| GSM2332168 | 2 |
| GSM2332169 | 3 |
| GSM2332170 | 4 |
| GSM2332171 | 2 |
| GSM2332172 | 3 |
| GSM2332173 | 3 |
| GSM2332174 | 2 |
| GSM2332175 | 3 |
| GSM2332176 | 3 |
| GSM2332177 | 3 |
| GSM2332178 | 2 |
| GSM2332179 | 2 |
| GSM2332180 | 3 |
| GSM2332181 | 5 |
| GSM2332182 | 2 |
| GSM2332183 | 2 |
| GSM2332184 | 5 |
| GSM2332185 | 2 |
| GSM2332186 | 3 |
| GSM2332187 | 5 |
| GSM2332188 | 3 |
| GSM2332189 | 2 |
| GSM2332190 | 3 |
| GSM2332191 | 5 |
| GSM2332192 | 3 |
| GSM2332193 | 5 |
| GSM2332194 | 2 |
| GSM2332195 | 2 |
| GSM2332196 | 1 |
| GSM2332197 | 3 |
| GSM2332198 | 2 |
| GSM2332199 | 5 |
| GSM2332200 | 5 |
| GSM2332201 | 2 |
| GSM2332202 | 2 |
| GSM2332203 | 3 |
| GSM2332204 | 4 |
| GSM2332205 | 2 |

**Table S8.** The performance of different combinations of two-gene ratio

|  | **Training** | | | **GSE83687** | | **GSE126124** | |
| --- | --- | --- | --- | --- | --- | --- | --- |
|  | Sensitivity | Specificity | Cutoff Value | Sensitivity | Specificity | Sensitivity | Specificity |
| PRLR/FGR | 93.90% | 88.10% | 0.885 | 60% | 100% | 0% | 100% |
| **PRLR/TNFSF13B** | **91.50%** | **85.70%** | **0.850** | **90%** | **77.80%** | **100%** | **83.30%** |
| SLC39A5/FGR | 90.20% | 95.20% | 1.052 | 90% | 88.80% | 100% | 8.30% |
| SC39A5/TNFSF13B | 86.50% | 93.90% | 0.996 | 66.70% | 100% | 0% | 100% |

**Table S9.** Immunofluorescence signal positive rates of PRLR and TNFSF13B and ratios

| **PRLR** | **TNFSF13B** | **Ratio** | **Patients** | **Subtype** |
| --- | --- | --- | --- | --- |
| 2.591 | 0.596 | 4.34732 | CT1 | Normal |
| 1.562 | 0.415 | 3.76386 | CT1 | Normal |
| 2.236 | 0.388 | 5.76289 | CT1 | Normal |
| 2.599 | 1.346 | 1.93091 | CT2 | Normal |
| 2.38 | 1.571 | 1.51496 | CT2 | Normal |
| 1.223 | 1.427 | 0.85704 | CT2 | Normal |
| 0.38 | 1.293 | 0.29389 | PT3 | WIA |
| 0.218 | 1.572 | 0.13868 | PT3 | WIA |
| 0.743 | 1.49 | 0.49866 | PT3 | WIA |
| 0.127 | 0.09 | 1.41111 | PT6 | WIA |
| 0.086 | 0.422 | 0.20379 | PT6 | WIA |
| 0.17 | 0.616 | 0.27597 | PT6 | WIA |
| 0.381 | 0.072 | 5.29167 | PT4 | IHL |
| 0.139 | 0.034 | 4.08824 | PT4 | IHL |
| 0.039 | 0.049 | 0.79592 | PT4 | IHL |
| 0.206 | 0.229 | 0.89956 | PT1 | WIA |
| 0.046 | 0.425 | 0.10824 | PT1 | WIA |
| 0.026 | 0.573 | 0.04538 | PT1 | WIA |
